# PKCα-Specific Phosphorylation of the Troponin Complex in Human Myocardium: A Functional and Proteomics Analysis

**DOI:** 10.1371/journal.pone.0074847

**Published:** 2013-10-07

**Authors:** Viola Kooij, Pingbo Zhang, Sander R. Piersma, Vasco Sequeira, Nicky M. Boontje, Paul J. M. Wijnker, Connie R. Jiménez, Kornelia E. Jaquet, Cris dos Remedios, Anne M. Murphy, Jennifer E. Van Eyk, Jolanda van der Velden, Ger JM. Stienen

**Affiliations:** 1 Laboratory for Physiology, Institute for Cardiovascular Research, VU Medical Center, Amsterdam, The Netherlands; 2 Johns Hopkins Bayview Proteomics Center, Department of Medicine, School of Medicine, Johns Hopkins University, Baltimore, Maryland, United States of America; 3 OncoProteomics Laboratory, Department of Medical Oncology, VU Medical Center, Amsterdam, The Netherlands; 4 St Josef-Hospital/Bergmannsheil, Clinic of the Ruhr-University of Bochum, Bochum, Germany; 5 Muscle Research Unit, Institute for Biomedical Research, The University of Sydney, Sydney, Australia; 6 Institute of Molecular Cardiobiology, Department of Pediatrics, School of Medical, Johns Hopkins University, Baltimore, Maryland, United States of America; 7 Department of Physics and Astronomy, VU University, Amsterdam, The Netherlands; Loyola University Chicago, United States of America

## Abstract

**Aims:**

Protein kinase Cα (PKCα) is one of the predominant PKC isoforms that phosphorylate cardiac troponin. PKCα is implicated in heart failure and serves as a potential therapeutic target, however, the exact consequences for contractile function in human myocardium are unclear. This study aimed to investigate the effects of PKCα phosphorylation of cardiac troponin (cTn) on myofilament function in human failing cardiomyocytes and to resolve the potential targets involved.

**Methods and Results:**

Endogenous cTn from permeabilized cardiomyocytes from patients with end-stage idiopathic dilated cardiomyopathy was exchanged (∼69%) with PKCα-treated recombinant human cTn (cTn (DD+PKCα)). This complex has Ser23/24 on cTnI mutated into aspartic acids (D) to rule out *in vitro* cross-phosphorylation of the PKA sites by PKCα. Isometric force was measured at various [Ca^2+^] after exchange. The maximal force (F_max_) in the cTn (DD+PKCα) group (17.1±1.9 kN/m^2^) was significantly reduced compared to the cTn (DD) group (26.1±1.9 kN/m^2^). Exchange of endogenous cTn with cTn (DD+PKCα) increased Ca^2+^-sensitivity of force (pCa_50_ = 5.59±0.02) compared to cTn (DD) (pCa_50_ = 5.51±0.02). In contrast, subsequent PKCα treatment of the cells exchanged with cTn (DD+PKCα) reduced pCa_50_ to 5.45±0.02. Two PKCα-phosphorylated residues were identified with mass spectrometry: Ser198 on cTnI and Ser179 on cTnT, although phosphorylation of Ser198 is very low. Using mass spectrometry based-multiple reaction monitoring, the extent of phosphorylation of the cTnI sites was quantified before and after treatment with PKCα and showed the highest phosphorylation increase on Thr143.

**Conclusion:**

PKCα-mediated phosphorylation of the cTn complex decreases F_max_ and increases myofilament Ca^2+^-sensitivity, while subsequent treatment with PKCα in situ decreased myofilament Ca^2+^-sensitivity. The known PKC sites as well as two sites which have not been previously linked to PKCα are phosphorylated in human cTn complex treated with PKCα with a high degree of specificity for Thr143.

## Introduction

Protein kinase C (PKC) is a member of the serine/threonine protein kinase family and is expressed in most tissues, including the heart. PKC is able to modify cardiac function via phosphorylation of proteins involved in calcium handling and in the regulation of contractile proteins. The thin filament proteins troponin T (cTnT) and I (cTnI) [1] and the thick filament proteins, myosin binding protein C (cMyBP-C) [1], myosin light chain 2 [2] as well as titin [3] are all known PKC targets. The members of the PKC family are differently expressed among species and co-localize with different target proteins within cardiomyocytes, making the full characterization of the effects of activation of PKC in human myocardium a daunting task. The PKC isoforms α, β, δ and ε have been implicated to play a critical role in the failing and hypertrophic heart [4], [5]. However, especially PKCα has been viewed as potential therapeutic target since its activity and expression increases in many models of cardiac injury, hypertrophy and failure and it is one of the predominant isoforms in the myocardium [4]–[9].

The troponin complex is an important substrate of PKCα and both positive and negative inotropic and lusitropic effects have been reported (reviewed by Metzger et al. 2004 and Layland et al. 2005) [10], [11]. Our previous studies revealed a decrease in Ca^2+^-sensitivity of force upon incubation of single permeabilized human cardiomyocytes with the catalytic subunit of PKC, as well as with the PKCε and PKCα isoforms, while no changes in isometric force at saturating Ca^2+^-concentration were observed [1], [12]. Direct application of PKCα to permeabilized human myocytes showed phosphorylation of cTnT and cTnI, but also of cMyBP-C, making it impossible to establish the functional consequences of PKCα-mediated phosphorylation of the troponin complex.

Known PKC phosphorylation targets include on cTnI: Ser42, Ser44, Ser76 (or Thr77) and Thr143 and on cTnT: Ser1, Thr194, Ser198, Thr203 and Thr284 (human sequence, cTnT isoform 3) [13]–[15]. Site-specific effects of phosphorylation on contractile properties have been reported, mostly by using transgenic animals with cTn phosphorylation mimicking charge mutations. Phosphorylation of Ser42 and/or 44 on cTnI has been shown to result in a reduction in both maximal force and Ca^2+^-sensitivity [16]–[18]. In contrast, phosphorylation of Thr143 on cTnI has been associated with sensitization of the myofilaments to Ca^2+^ [19]. In addition, Sumandea et al. [20] reported that Thr206 in mice, which corresponds with Thr203 in human cTnT isoform 3, is a functionally critical cTnT PKC phosphorylation residue. Pseudo-phosphorylation at this cTnT site resulted in a significant reduction of maximal isometric tension and Ca^2+^-desensitization of force. So far, the site-specific effects in human tissue remain illusive. Therefore, current study aimed to investigate the specific effects of human cTn phosphorylation by PKCα on contractility, and to explore which phosphorylation targets might be involved.

The specific role of PKCα-mediated phosphorylation of cTn in cardiomyocytes was analyzed in myocardium from end-stage heart failure patients using our previously described cTn exchange method [21]. This method allows determination of the direct effects of PKCα-mediated cTn phosphorylation on contractility in human cardiac preparations without altering the phosphorylation status of other contractile proteins. PKCα-treated cTn complex was exchanged in failing tissue in which the endogenous cTnI phosphorylation levels are low. In the recombinant cTn complex the protein kinase A (PKA) sites on cTnI Ser23/24 were mutated into aspartic acids (cTn (DD)) to rule out *in vitro* cross-phosphorylation of these PKA sites by PKCα. To identify the origin of the changes in contractile function observed, we investigated the targets of PKCα in human cTn using site-specific phospho-antibodies, liquid chromatography (LC) MS/MS and a targeted MS-based method, multiple reaction monitoring (MRM), which allows quantitation of site-specific phosphorylation.

Our results revealed that exchange using PKCα-treated cTn (DD) resulted in a sensitization of the myofilaments to Ca^2+^ but a depression of the maximal force generating capacity (F_max_) of cardiomyocytes. The overall effects of PKCα-mediated phosphorylation of the cTn complex on cardiomyocyte force development were negative. In contrast to the Ca^2+^-sensitizing effect of the PKCα-treated cTn (DD) complex, subsequent PKCα-incubation of the cardiomyocytes after exchange resulted in a desensitization of the myofilaments to Ca^2+^. This indicates that PKCα-mediated phosphorylation of sarcomeric proteins other than cTn exerts opposing effects on Ca^2+^-sensitivity. LC MS/MS analysis of human recombinant cTn complex treated with PKCα revealed two sites that, for the first time, are identified as PKCα substrates: Ser198 located on cTnI and Ser179 on cTnT. In addition, MRM analysis revealed target-specificity in the *in vitro* PKCα-mediated phosphorylation of Ser42, Ser44, Thr143, and Ser198 on cTnI.

## Materials and Methods

An expanded methods section is available in File S1.

### Phosphorylation of human recombinant cTn with PKCα and protein analysis

Human recombinant cTn complex was prepared as described before [22]. Recombinant cTn complex in which the PKA sites Ser23/24 were mutated into aspartic acid (D) was used to rule out cross-phosphorylation of these sites by PKCα that occurs *in vitro* but not *in vivo* (Figure S1) [1]. Cardiac Tn (DD) complex was maximally phosphorylated by human recombinant PKCα isozyme (Sigma, P1782). Thereto, the cTn complex was incubated with PKCα (24 μg/ml PKCα, 1 mmol/L Na_2_ATP (sodium adenosine-5′-triphosphate), 4 mmol/L MgCl_2_, 6 mmol/L DTT, 10 μmol/L PMA (phorbol 12-myristate 13-acetate; Sigma), 10 μl/ml phosphatase inhibitor cocktail (PhIC, Sigma, P5726), and 5 μl/ml protease inhibitor cocktail (PIC, Sigma, P8340) for 180 minutes at 30°C. The phosphorylated cTn (DD) complex (cTn (DD+PKCα)) was dialyzed overnight in order to remove ATP. Samples were taken at different time–points to assess the time course of phosphorylation.

Analysis of PKCα-mediated phosphorylation of recombinant cTn (DD) complex proteins was determined using ProQ Diamond stained (Molecular Probes) 1D gradient gels and Western blotting using antibodies against cTnI Ser42 and Thr143 (Abcam) as described previously [12].

### Exchange of cardiac troponin complex in failing human cardiomyocytes

In the exchange experiments, left ventricular samples (*n* = 6) from end-stage failing idiopathic dilated myocardium (IDCM, NYHA Class III and IV, table 1) were used. Patient details are shown in Table 1. Samples were obtained after written informed consent and with approval of the local Ethical Committees (Human Research Ethics approval from St. Vincent's Hospital (H03/118) and from The University of Sydney (#7326)). The investigation conforms to the principles outlined in the Declaration of Helsinki. Single cardiomyocytes were isolated, Triton X-100 permeabilized and exchanged with recombinant cTn complex as described before with a few adjustments [21]. In short, single cardiomyocytes were mechanically isolated in ice-cold rigor solution (132 mmol/L NaCl, 5 mmol/L KCl, 1 mmol/L MgCl_2_, 10 mmol/L Tris, 5 mmol/L EGTA, 1 mmol/L NaAzide, pH 7.1) and permeabilized by addition of 0.5% Triton X-100 for 5 minutes [23], [24]. After permeabilization, cells were washed twice with rigor solution and finally washed in exchange solution (10 mmol/L imidazole, 5 mmol/L MgCl_2_, 3 mmol/L CaCl_2_, 2.5 mmol/L EGTA, pH 6.9). Single cardiomyocytes were exchanged with recombinant cTn complex as described before [21].

**Table 1:** Patient characteristics.

Patient	Gender	Age	NYHA Class	LVEF (%)
1	F	50	III	33
2	M	53	III	25
3	F	52	III	15
4	M	53	IV	15
5	M	58	IV	15
6	M	62	III/IV	23

LVEF, LV ejection fraction; F, Female; M, Male. Medication used included angiotensin-converting-enzyme inhibitors, angiotensin II receptor antagonists, β-blockers, digoxin and anti-arrhythmic agents.

### Isometric force measurements in single human cardiomyocytes

Force measurements on permeabilized cardiomyocytes were performed as described previously [22], [24]. Sarcomere length was adjusted to 2.2 μm and force measurements were performed at 15°C. After an initial series of measurements at different Ca^2+^-concentrations, myocytes were incubated with activated PKCα and subsequently force measurements were repeated. To measure the K_tr_, after reaching steady force, the cardiomyocyte was 20% reduced in length within 2 ms and restretched after 30 ms (slack test). During this slack test, force first dropped to zero and after the restretch quickly redeveloped to the original steady state level. A single exponential was fitted to estimate the rate constant of force redevelopment at maximal activation (Ktr-max).

### LC MS/MS analysis of human recombinant cTn complex incubated with PKCα

Coomassie-stained protein bands were excised and processed for trypsin in-gel digestion according to the protocol of Gundry et al. [25]. The LC-MS/MS analysis and database searching was performed as described in File S1.

### MRM MS assay of PKCα-treated donor and failing tissue and human recombinant cTn

Tissue from end-stage heart failure was incubated with PKCα as described before [1]. A mass spectrometry based method, MRM analysis was designed to determine the fold increase of phosphorylation of Ser198 on cTnI in control (untreated tissue) and PKCα-treated tissue from donor and heart failure patients (*n* = 4 per group; technical replicates  = 3).

Human recombinant cTn was maximally phosphorylated by PKCα (based on the phospho specific Pro-Q Diamond staining) and MRM was used to determine PKCα induced phosphorylation levels of recombinant cTnI (Table S2). Analysis of Ser23/24 phosphorylation was excluded from our MRM analysis as these sites are charge mutated into aspartic acid and cannot be phosphorylated. Details of method and assay development are given in File S1.

### Data analysis

Myofilament data analysis was performed using the modified Hill equation to fit force-pCa relations. Comparisons between the cTn (DD) and cTn (DD+PKCα) groups were made using an unpaired Student *t*-test or ANOVA, where appropriate. Values are given as means ± S.E.M. of *n* myocytes.

MRM data acquisition, processing and analysis were performed using Applied Biosystems/MDS Sciex Analyst software and Multiquant 1.0. The average peak areas for each phosphorylated tryptic peptide were expressed in absolute quantities (fmol) using standard curves constructed using known amounts of synthetic peptides and the values obtained were corrected for loading differences by densitometric analysis of the bands excised from the SDS-PAGE gel (Figures S4 and S5).

## Results

### PKCα-mediated phosphoryation of cTn

**Figure 1:**
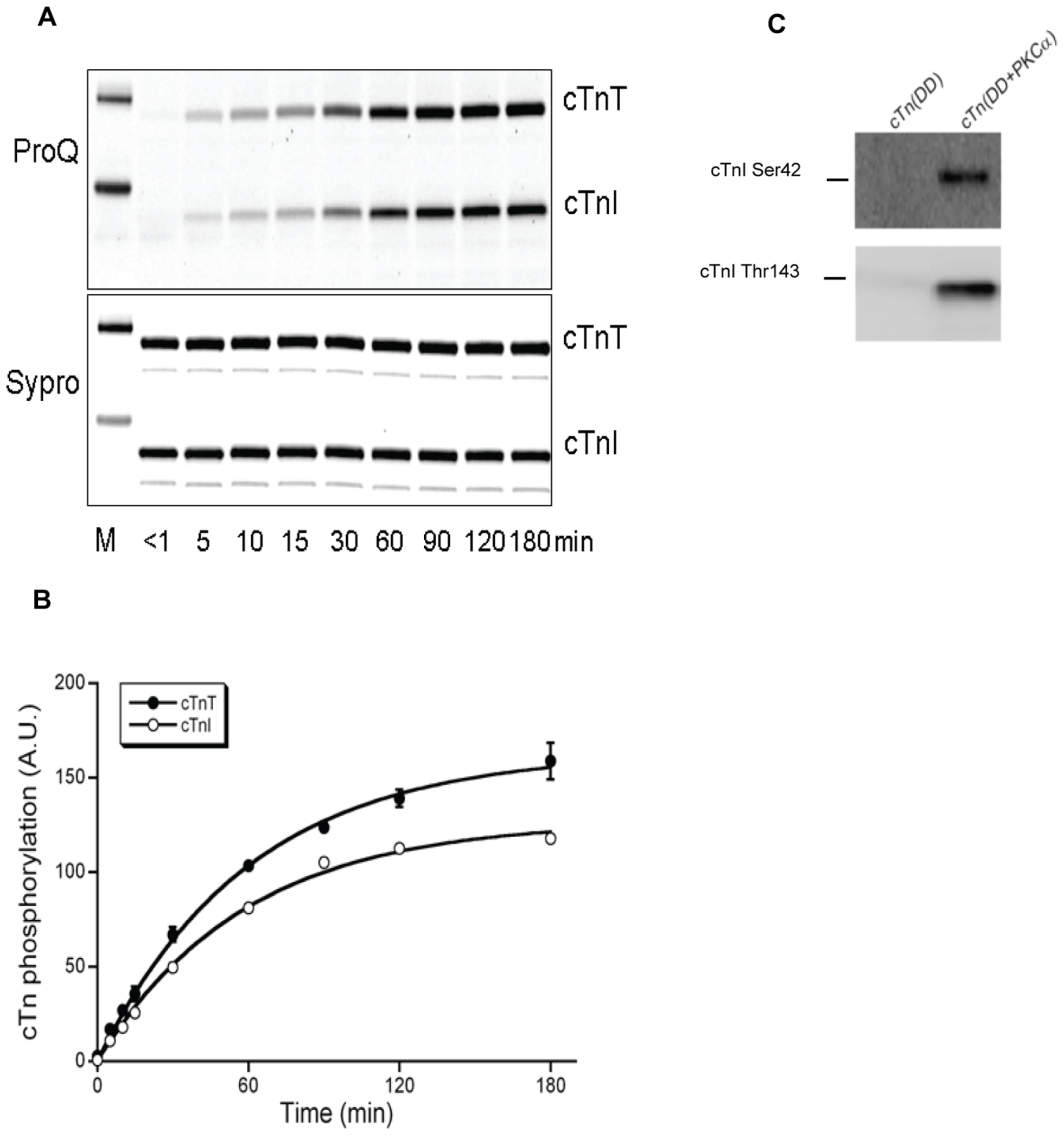
PKCα-mediated phosphorylation of human recombinant cTn complex (cTn (DD)). (**A**) Samples were taken at different time points and run on a 1D gradient gel stained with ProQ Diamond and with Sypro Ruby (M: Peppermint molecular weight marker). (**B**) Time course of PKCα-mediated phosphorylation of recombinant cTn (DD) complex. Mean values are shown (±S.E.M.) obtained from two different 1D-gels; the intensities of the cTnI bands on the Sypro Ruby stained gels were used to correct for loading differences. Time constant (±S.E.M.) of the exponential fitted to the data points: 60±3 minutes for cTnT and 58±4 minutes for cTnI phosphorylation. (**C**) Western blotting of the cTn (DD) complex incubated with PKCα for 90 minutes (cTn (DD+PKCα)) showed phosphorylation at cTnI sites Ser42 and Thr143 with phospho-specific antibodies.

Incubation of recombinant cTn (DD) complex with PKCα resulted in phosphorylation of both cTnT and cTnI. The ProQ Diamond stained gel shown in Figure 1A and B showed that cTnT and cTnI phosphorylation reached a steady state within the 3 hours of PKCα incubation. The final levels of phosphate incorporation reached were similar. The time constant of the exponential fitted to the data points amounted to approximately 1 hour in both cases and suggests that the overall affinities of PKCα for the subunits are similar. Western blotting with phospho-specific antibodies showed that Ser42 and Thr143 on cTnI (Figure 1C) were phosphorylated in cTn complex incubated with PKCα.

### PKCα-mediated phosphorylation of cTn increases Ca^2+^-sensitivity

Exchange experiments were conducted in tissue samples from 6 different end-stage failing hearts. The choice to use tissue from patients with end-stage heart failure instead of control tissue was based on our previous results where an effect of PKCα phosphorylation was only observed in the failing and not in the control group [12]. Moreover, PKCα is found to be up-regulated in the end-stage failing heart [9].

Previous results showed that with the experimental conditions used ∼69% of the endogenous cTn in permeabilized failing cardiomyocytes was exchanged by recombinant human cTn complex [21]. In order to verify whether this value also applied to the cTn (DD) complex used in the present study, we measured the amount of cTn (DD) exchange in control (donor) tissue. The percentage of exchange amounted to 69.4±13.3% (*n* = 3) (Figure S2), i.e. very similar to the value obtained before. To inhibit all proteases and phosphatases during the exchange of cTn, inhibitor cocktails were added.

To assess the effects of PKCα-mediated cTn phosphorylation on Ca^2+^-sensitivity of force measurements were performed at various [Ca^2+^] in single cardiomyocytes (*n* = 41). Exchange using 1.0 mg/ml unphosphorylated cTn (DD) complex (*n* = 34 myocytes) resulted in a significant decrease of the Ca^2+^-sensitivity compared to the time control cells (ΔpCa_50_ = 0.09±0.02), which were kept in exchange solution without cTn complex (Table 2).

**Table 2:** Overview of cardiomyocyte force measurements.

Failing Myocardium					
	F_max_	F_pas_	pCa_50_	nH	K_tr_-max
	6 hearts; 35 myocytes				
*Time control*	25.2±2.3	2.9±0.3	5.60±0.01	2.6±0.2	0.69±0.03
	6 hearts; 34 myocytes (5 hearts; 13 myocytes after incubation)				
*cTn (DD)*	26.1±1.9	2.3±0.3	5.51±0.02^†^	2.5±0.1	0.62±0.02
*+PKC*α *incubation*	22.4±2.2	2.0±0.3	5.43±0.02*	2.5±0.1	0.64±0.05
	5 hearts; 25 myocytes (4 hearts; 9 myocytes after incubation)				
*cTn (DD+PKC*α*)*	17.1±1.9^#^	2.7±0.3	5.59±0.02	2.8±0.1	0.67±0.04
*+PKC*α *incubation*	17.2±3.1	1.8±0.2	5.45±0.02*	2.4±0.1*	0.59±0.09

Time control denotes results obtained from cardiomyocytes kept in exchange buffer without cTn complex added. Symbols used: # denotes P<0.05, cTn (DD+PKCα) compared to time control or cTn (DD) and † denotes P<0.05, cTn (DD) compared to time control in ANOVA followed by Bonferroni post-hoc test. Measurements in cTn exchanged cells were repeated after treatment with PKCα. In this case * denotes P<0.05, before vs. after incubation with PKCα in paired *t*-test. Abbreviations: F_max_, maximal force per cross-sectional area at saturating calcium concen-tration (pCa4.5) in kN/m^2^; F_pas_, passive force per cross-sectional area in relaxing solution (pCa 9) in kN/m^2^; nH, steepness of the force-pCa curves; K_tr_-max, the rate of force redevelopment at maximal activating solution (pCa 4.5) in s^−1^.

Moreover, donor cardiomyocytes were exchanged with cTn (DD) complex, which showed no functional differences compared to control cardiomyocytes (Figure S3). This lack of effect of pseudophosphorylated cTn (DD) is as expected since non-failing donor samples show high cTnI phosphorylation at the PKA sites (Ser23/24) [21].

**Figure 2:**
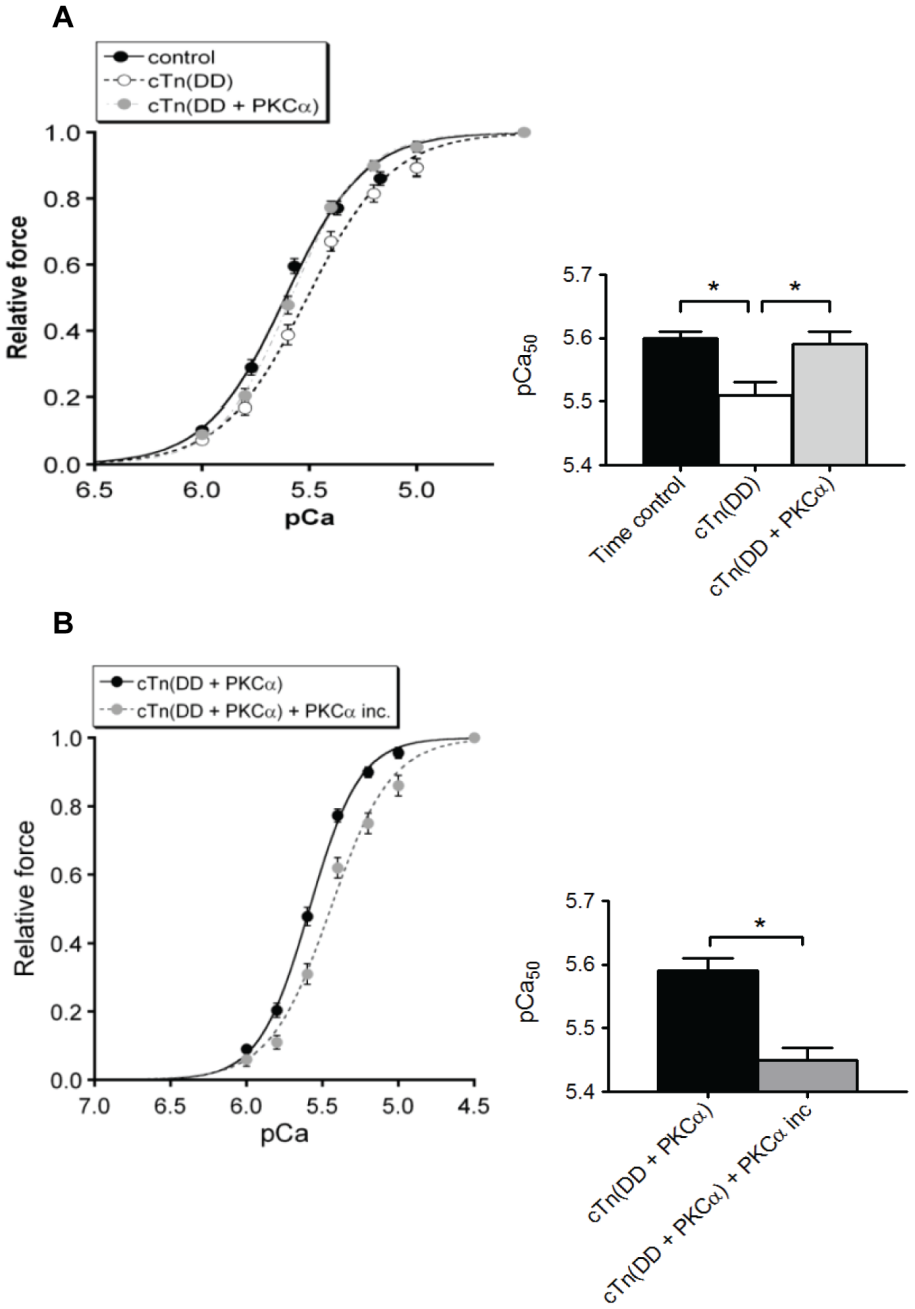
Effect of cTn (DD+PKCα) exchange on the Ca^2+^-sensitivity of force. (**A**) The Ca^2+^-sensitivity derived from the midpoint of the force-pCa relationship (pCa_50_) increased upon exchange with cTn (DD+PKCα) compared to failing cardiomyocytes exchanged with cTn (DD). *P<0.05, cTn (DD) exchange vs. cTn (DD+PKCα) exchange in ANOVA, followed by Bonferroni post-hoc test. Time control denotes results obtained from cardiomyocytes kept in exchange buffer without cTn complex added. (**B**) Subsequent incubation of PKCα of the cardiomyocytes exchanged with cTn (DD+PKCα) significantly reduced Ca^2+^-sensitivity of force development. *P<0.05 cTn (DD+PKCα) vs. cTn (DD+PKCα) + PKCα in paired *t*-test.

Figure 2A illustrates that exchange of PKCα-pretreated cTn complex (cTn (DD+PKCα)), which was treated to obtain maximal phosphorylation (180 minutes), significantly increased Ca^2+^-sensitivity compared to exchange with cTn (DD) (ΔpCa_50_ = 0.08±0.02). The cardiomyocytes exchanged with cTn (DD+PKCα) were subsequently incubated for 60 minutes with PKCα to determine the effects of additional PKCα-mediated protein phosphorylation as performed in a previous study [1]. During the PKCα incubation, Calyculin A, a serine/threonine protease inhibitor was added to prevent dephosphorylation.

These experiments revealed a significant *decrease* in myofilament Ca^2+^-sensitivity upon incubation of the cells with PKCα (ΔpCa_50_ = 0.14±0.03) (Figure 2B). This observation is in agreement with our previous observations [1]. It clearly shows that the direct effects of PKCα-mediated phosphorylation of the cTn complex probed by the exchange method are in contrast with the combined effects on Ca^2+^-sensitivity observed upon PKCα treatment of all contractile proteins within the myofilament lattice. Table 2 presents an overview of these results.

### PKCα-mediated phosphorylation of cTn depresses maximal force generation

**Figure 3:**
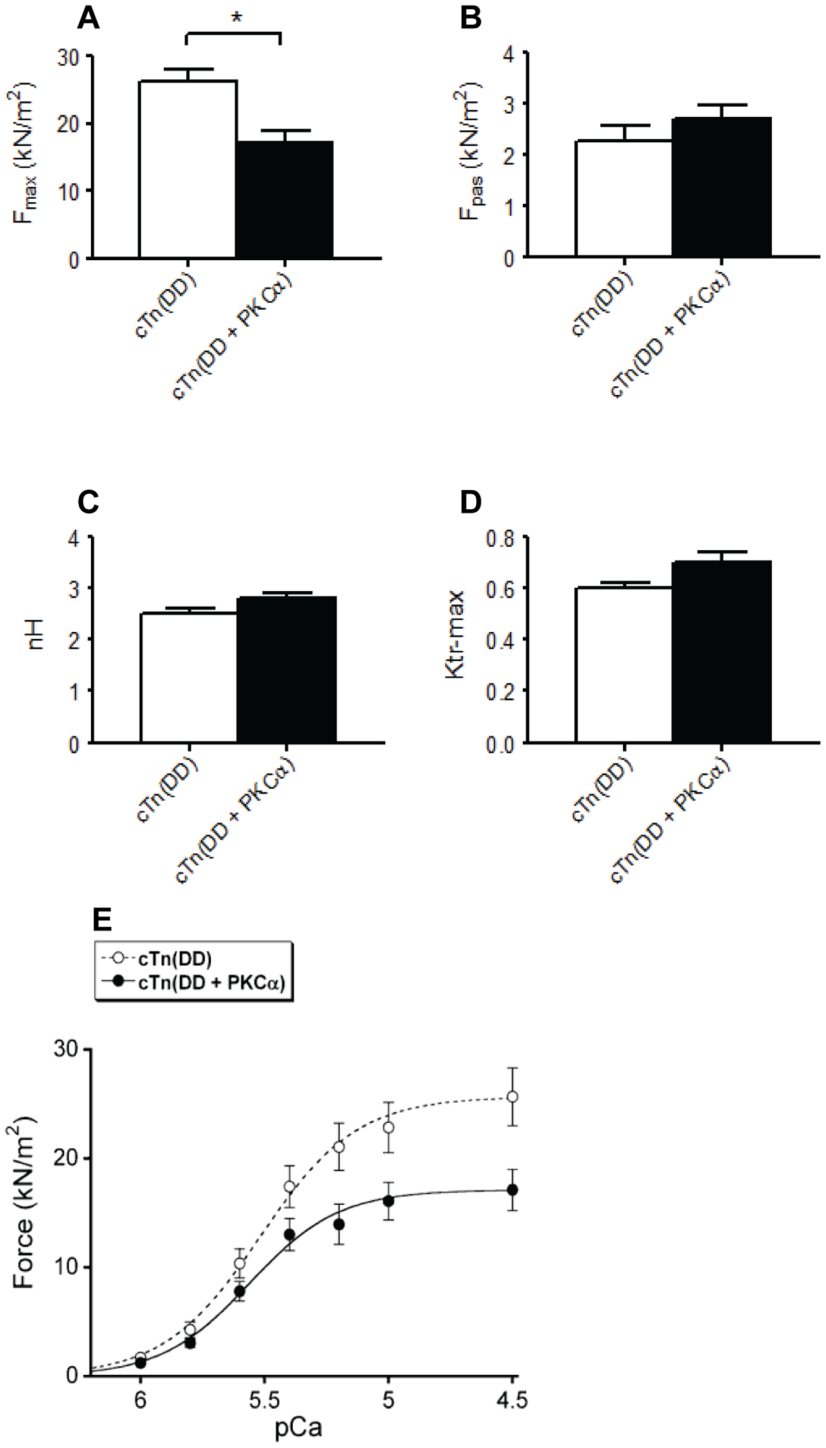
Effects of cTn (DD) and cTn (DD+PKCα) exchange in failing cardiomyocytes. (**A**) The maximal force generating capacity of the cardiomyocytes was significantly reduced in the cells exchanged with cTn (DD+PKCα) compared to the cTn (DD) group and the cTn (DD) group. *P<0.05, *t-*test. The passive force, as well as the steepness of the force–pCa relation (nH), and the rate of force redevelopment (K_tr_-max) were not significantly different among the groups (**B–D**). The maximum force at saturating Ca^2+^-concentration was significantly reduced in the cardiomyocytes exchanged with cTn (DD+PKCα). The combined effects of cTn exchange on maximal force and Ca^2+^-sensitivity resulted in a depressed force in the cTn (DD+PKCα) group compared to the cTn (DD) group at maximal and submaximal Ca^2+^ concentrations (* P<0.05 in *t*-test) (**E**).

Exchange of endogenous cTn with cTn (DD) did not change the maximal isometric force (F_max_) at saturating Ca^2+^ concentration (pCa 4.5) (Table 2). However, cardiomyocytes exchanged with cTn (DD+PKCα) showed a significant reduction in F_max_ compared to time control cells (Table 2) and cells exchanged with cTn (DD) (Figure 3A). The combined effects of the changes in relative force and pCa_50_ values are shown in Figure 3E. This figure illustrates that the isometric force (force/cross-sectional area) is depressed at all Ca^2+^ concentrations in the cTn (DD+PKCα) group compared to the cTn (DD) group. Thus, the cumulative effect of PKCα-mediated phosphorylation of cTnI and cTnT on F_max_ and pCa_50_ is negative.

To determine if PKCα-mediated phosphorylation lowers maximal force in human cardiomyocytes after exchange with cTn (DD) additional force measurements were performed before and after (1 hour) incubation with PKCα. These results did not show a decrease in F_max_ after incubation with PKCα (F_max_ = 22.4±2.2 kN/m^2^) compared to the F_max_ before incubation (F_max_ = 25.2±3.1 kN/m^2^) (P = 0.08, *n* = 13) (Table 2). Myocyte measurements showed that prolongation of the incubation time to 3 hours did not have a significant effect on Fmax. This suggests that *in vitro* phosphorylation of the cTn complex prior to exchange is required to observe an effect of PKCα on F_max_.

### PKCα-mediated phosphorylation of cTn has no effect on other contractile properties

Exchange of endogenous cTn with cTn (DD) did not change passive force (F_pas_) measured at pCa 9 compared to untreated time control cells (Table 2). In addition, F_pas_ did not significantly differ between cTn (DD) and cTn (DD+PKCα) (Figure 3B). Recently, PKCα-mediated phosphorylation of titin has been shown to increase passive stiffness [3]. However, subsequent PKCα treatment of cTn exchanged cardiomyocytes did not significantly affect F_pas_.

In addition, no effect of cTn (DD) exchange was seen on the steepness of the force-pCa relation (nH) or the rate of force redevelopment (K_tr_-max) measured at pCa 4.5 after a slack test (Table 2). Furthermore, no significant differences were observed in nH and K_tr_-max upon exchange with cTn (DD+PKCα) compared to cTn (DD) (Fig. 3C,D). PKCα treatment of cTn exchanged cardiomyocytes did not alter nH or K_tr_-max (Table 2). This suggests that PKCα-mediated phosphorylation of cTn or of any other sarcomeric protein has no effect on cross-bridge kinetics.

### LC MS/MS analysis

**Figure 4:**
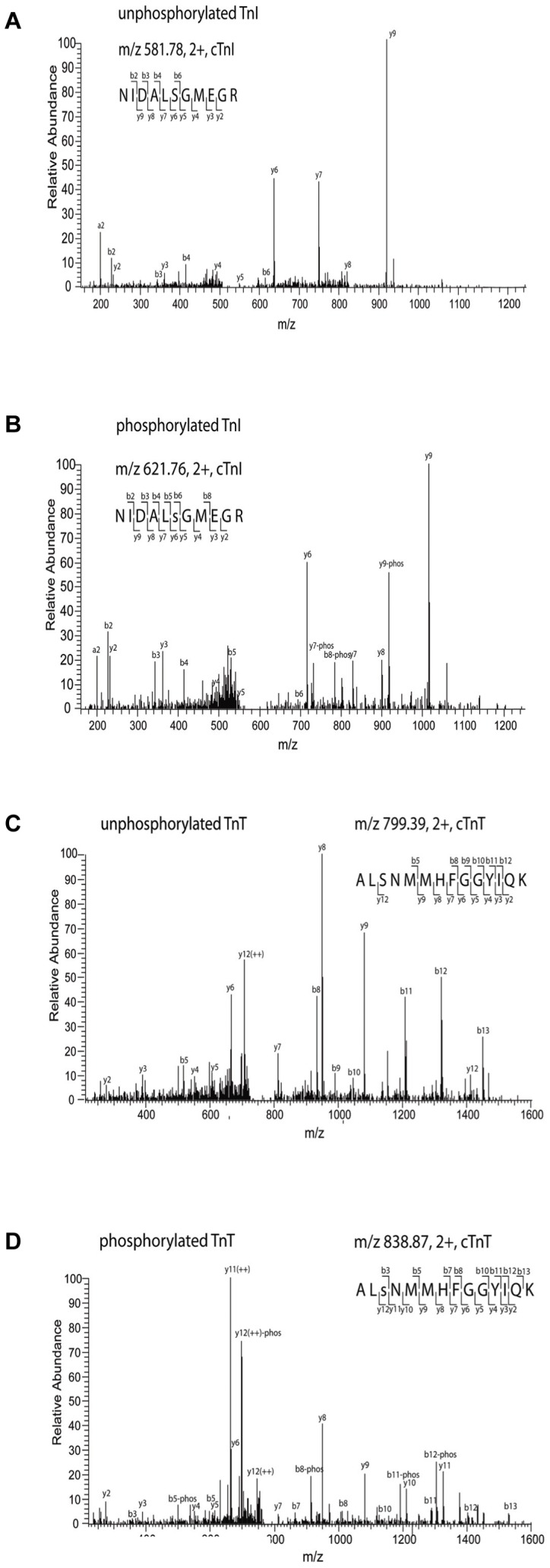
Novel identified PKCαphosphorylation sites on cTnI and cTnT: Ser198 on cTnI and Ser179 on cTnT identified in human recombinant cTn complex. (**A**) MS/MS spectrum of the m/z 581.78 unphosphorylated peptide (**B**) MS/MS spectrum of the m/z 621.76 phosphorylated peptide. (**C**) MS/MS spectrum of the m/z 799.39 unphosphorylated peptide (**D**) MS/MS spectrum of the m/z 838.87 phosphorylated peptide. The b-ions denote N-terminal ions and y-ions denote C-terminal ions. The tandem MS (MS/MS) of our phosphorylated peptide ions undergo a preferential neutral loss of H_3_PO_4_ (98 Da). The MS/MS patterns confirm the phosphorylation of the two peptides and identify the novel phosphorylation sites.

For LC MS/MS analysis, PKCα-treated recombinant cTn was digested using trypsin immediately after mixing of the solutions at time-points “0” (<1 min) and after 180 minutes of incubation. The amino acid sequence coverage of cTnT and cTnI was on average 47% and 40%, respectively. Both the phosphorylated and unphosphorylated cTnI peptides of NIDALsGMEGR ([M+2H]^2+^, m/z 621.76; M+2H]^2+^, m/z 581.78, respectively) comprising residues 193–203 were observed in the PKC treated sample (spectrum see Figure 4A,B) indicating that Ser198 is phosphorylated. Furthermore, the PKCα-treated cTnT, peptide ALsNMMHFGGYIQK ([M+2H]^2+^, m/z 838.87), and that of the unphosphorylated peptide ([M+2H]^2+^, m/z 799.39) comprised residues 177–188 identified Ser179 as a target for PKCα (Figure 4C,D).

### MRM MS assay of PKCα-treated failing tissue and human recombinant cTn

**Figure 5:**
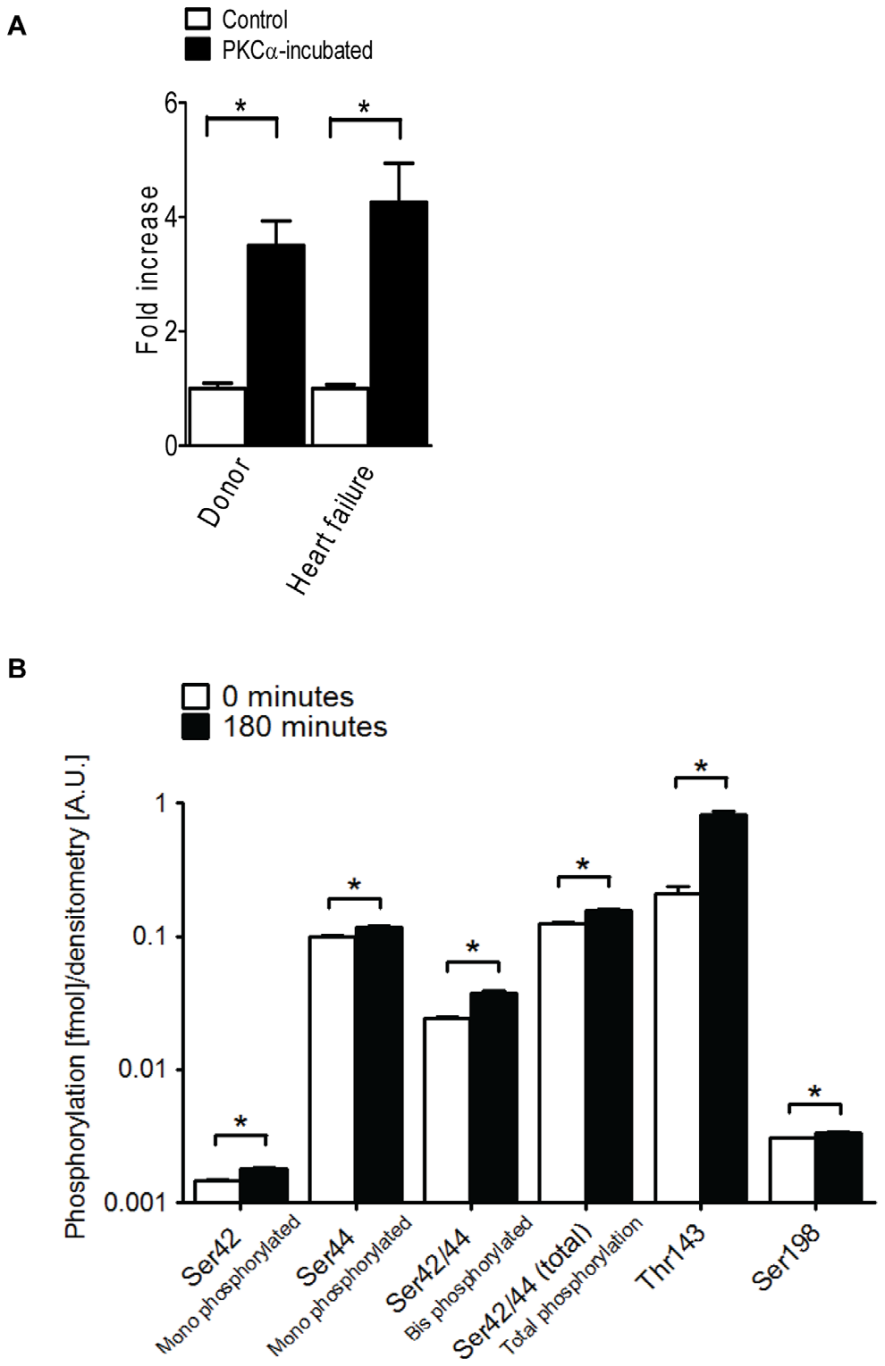
Site-specific quantification of PKCα-treated human donor and failing tissue and recombinant cTnI using MRM. A . The fold increase of phosphorylation of Ser198 on cTnI in Control (untreated tissue) and PKCα-treated donor and failing tissue (*n* = 4 per group; technical replicates  = 3). All values from the tissue samples were determined using synthesized internal standard peptides, and expressed relative to total cTnI content. Error bars indicate the standard error of mean (SEM). * *P*<0.005 in unpaired student *t*-test **B**. To quantify the phosphorylation status of each site in recombinant cTnI incubated with PKCα, MRM assays were designed for each mono or di-phosphorylated sequence and analyzed (technical replicates  = 4). The obtained values were corrected for loading differences by the intensity of the coomassie-stained excised gel bands. A significant increased phosphorylation was observed for all the phosphorylation sites after PKCα-treatment. A zoom-in of the Ser198 bar has been inserted on the side for clarification. *P<0.05, time-point <1 minute vs. time-point 180 minutes in paired *t*-test.

Figure 5a shows the phosphorylation increase of amino acid residue Ser198 upon PKCα-treatment. Donor tissue showed a fold increase of 3.50±0.43 (*n* = 4; technical replicates  = 3) and failing tissue a fold increase of 4.26±0.68 (*n* = 4; technical replicates  = 3) after PKCα treatment. This indicates that cTnI Ser198 is a substrate for PKCα in both donor and failing tissue.

Site-specific quantification of PKCα-treated human recombinant cTnI was performed using MRM analysis against Ser42, Ser44, Thr143 and Ser198. Synthetic peptides corresponding to unmodified and modified forms of each cTnI tryptic fragment containing the PKC phosphorylatable residues (Table S1) were used to create standard curves based on six-point dilutions of synthesized and labeled internal standard phosphorylated peptides IsASR, ISAsR, IsAsR, RPtLR, and NIDALsGMEGR (Figure S6). The labeled peptide has a N15 incorporated at the C-terminus. The known PKC phosphorylatable sites Ser42, Ser44, diphosphorylated Ser42/44, Thr143 and the novel site, Ser198 were quantified at t <1 min and after 180 minutes of incubation by PKCα. Thr143 showed the largest amount of PKCα-induced phosphorylation (0.822±0.042). PKCα-induced phosphorylation of Ser44 was intermediate (0.116±0.002) and that of Ser42 (0.00178±0.00004), Ser42/44 (0.037±0.002) and Ser198 (0.00336±0.00001) (Figure 5B, Table S2) was relatively low. Note that for t<1 min, part of the sites are already phosphorylated showing that initial cTnI phosphorylation might be fast.

## Discussion

This study aimed to investigate the functional effects of PKCα-mediated phosphorylation of the troponin complex in human cardiomyocytes and to resolve the targets involved. The main finding from the cTn exchange experiments was that specific PKCα-mediated phosphorylation of the troponin complex *in vitro* resulted in an increase in Ca^2+^-sensitivity and a reduction in the force generating capacity. Conversely, PKCα treatment after exchange resulted in a decrease in Ca^2+^-sensitivity, most likely via phosphorylation of other targets within the myofilament lattice. Moreover we identified two PKCα phosphorylation substrates on human cTn: Ser198 located on cTnI and Ser179 on cTnT, which have not previously been linked to PKC and provided evidence of target specificity in the phosphorylation of cTnI.

### Specific PKCα-mediated phosphorylation of troponin increases myofilament Ca^2+^-sensitivity

Our results showed an increase in myofilament Ca^2+^-sensitivity in human cardiomyocytes exchanged with cTn (DD+PKCα) when compared to the cTn (DD) group. This indicates that there are specific sites on cTnT and/or cTnI phosphorylated by PKCα that enhance myofilament Ca^2+^-sensitivity. Previously, Wang et al. [19] reported an increase in myofilament Ca^2+^-sensitivity after PKC-βII application by direct phosphorylation of cTnI at Thr143, a unique residue in the inhibitory region of cTnI. Our results (Figure 1) show that indeed Thr143 was phosphorylated by PKCα and Figure 5 identified this site as the major PKCα phosphorylation site on cTnI. PKCα also phosphorylated Ser42 and Ser44 on cTnI in the recombinant cTn (DD) complex (Figures 1 and 5), although to a lesser extent than Thr143. Pseudo-phosphorylation of Ser43 and Ser45 (equivalent to Ser42 and Ser44 in human) on cTnI in mice desensitized myofilaments to Ca^2+^ [16]. Taken together, these data suggest that cTnI phosphorylation of Thr143, but not Ser42 and Ser44 probably underlies the observed increase in Ca^2+^-sensitivity.

Jideama et al. reported a reduction in myofilament Ca^2+^-sensitivity upon phosphorylation of cTnT by PKCα [13]. Previously, we observed increased myofilament Ca^2+^-sensitivity upon dephosphorylation of cTnT by alkaline phosphatase which is in agreement with these findings [1]. It must be noted, however, that Jideama et al. also reported PKCζ phosphorylation of two unknown sites on cTnT, which resulted in an increase in Ca^2+^-sensitivity [13]. Possibly PKCα phosphorylates the PKCζ sites in the recombinant cTn complex and this might underlie the observed increase in myofilament Ca^2+^-sensitivity. Interestingly, our novel identified phosphorylation cTnT site, Ser179, might be one of the previously unidentified PKCζ sites [13].

Thus, even though phosphorylation of Ser43 and Ser45 on cTnI (mouse sequence) have been shown to reduce the Ca^2+^-sensitivity of force, our study shows that the net result of phosphorylation of cTnI and/or cTnT by PKCα is an increased sensitivity of the myofilaments for Ca^2+^. It should be noted that the phosphorylation level of site Thr143 is approximately 5 times higher than phosphorylation of Ser42/44 after PKCα incubation. This preference of PKCα could be the cause of a dominant effect of Thr143 phosphorylation over total Ser42/44 phosphorylation.

PKCα-treatment after exchange resulted in a decrease in Ca^2+^-sensitivity, which is in agreement with our earlier findings in failing cardiomyocytes incubated with PKCα [1]. In principle this could be caused by phosphorylation of other targets within the myofilament lattice (including those on the 30% endogenous complex remaining after the exchange) or by additional phosphorylation of the cTn complex, including sites not accessible *in vitro*. Apart from cTnI and cTnT also cMyBP-C and titin can be phosphorylated by PKCα. On cMyBP-C are sites Ser275 and Ser304 (human sequence) identified as PKC substrates [26]–[28], yet the effects of PKCα-mediated phosphorylation of cMyBP-C on contractility are unclear. Ser170 and Ser26 in the PEVK region of titin have recently been identified as PKCα substrates [3]. Whether PKCα-mediated phosphorylation of titin might influence myofilament Ca^2+^-sensitivity of force in human cardiomyocytes remains to be established.

We show that the newly identified site on cTnI, Ser198, is phosphorylated to some extent in failing myocardium where it is a substrate of PKCα and, in particular, that Thr143 is the preferred substrate for PKCα on cTnI. Therefore, phosphorylation of Thr143 is likely to play a major role in the observed effects but caution should be exerted when extrapolating these data to the *in vivo* situation. It can be noted that the extent of phosphorylation of the different phosphorylation sites as observed by Zhang et al. [29] in human cardiac tissue is comparable to the phosphorylation levels we observed in recombinant cTn. These authors measured Thr143 as highest phosphorylated site on cTnI, which is in agreement with our results (Figure 3). Furthermore, we found that exchange of PKCα phosphorylated cTn complex resulted in an increase in Ca^2+^-sensitivity of force and a reduction in the maximal force generating capacity (F_max_) and that the combined effect on force development is negative. Subsequent incubation of the cardiomyocytes with PKCα resulted in a decrease in Ca^2+^-sensitivity but did not affect F_max_. While the increase in Ca^2+^-sensitivity is due to phosphorylation of sites on cTn, we attribute the decrease in Ca^2+^-sensitivity after subsequent incubation with PKCα to phosphorylation of one or more myofilament protein (s) other than cTn.

Van der Velden et al. showed that the effect of PKC phosphorylation on Ca^2+^-sensitivity is greater in failing than in healthy control cardiomyocytes even though it has been shown that the expression and activity is upregulated in many models of cardiac injury, hypertrophy and failure [4]–[9], [12]. This discrepancy might be explained by the localized action of PKC isoforms or by differences in the kinase/ phosphatase balance between healthy and diseased hearts.

### Specific PKCα-mediated phosphorylation of troponin reduces the maximal force generating capacity

Exchange of endogenous cTn in failing cardiomyocytes with cTn (DD+PKCα) complex decreased the maximal force generating capacity of the failing cardiomyocytes. These findings resemble the results of Belin et al. in rat skinned myocytes in which a marked depression of the F_max_ was observed upon treatment with recombinant PKCα [9]. The depressive effect of PKCα on F_max_ was only observed in healthy tissue and not in failing rat myocardium [9]. Several studies in rodents reported that phosphorylation of Ser43 and Ser45 (mouse sequence) on cTnI by PKC reduces the maximal force generating capacity of cardiac muscle cells [16]–[18]. Since we observed phosphorylation of Ser42 and Ser44 by PKCα in the human cTn complex (Figures 1 and 5) it is possible that phosphorylation of these sites underlies the decreased maximal force. The difference in the impact of PKCα on F_max_ in failing tissue in the study of Belin et al. and in our study could imply that the relevant site in their study in rats was already saturated, whereas this was not the case in our failing human samples.

The reduction in maximal force was not observed in failing cardiomyocytes that were incubated with PKCα. Moreover, the decreased F_max_ in the cTn (DD+PKCα) group could not be reduced further nor could it be corrected by subsequent incubation with PKCα. One explanation for the lack of a decrease in maximal force in the human failing myocytes directly incubated with PKCα might be that certain PKC sites (eg. Ser42/44 on cTnI and Thr203 on cTnT) are only exposed and sufficiently phosphorylated in recombinant cTn protein but not when the cTn complex is part of the intact filaments. Alternatively, coincident phosphorylation of other myofilament proteins (eg. cMyBP-C) may exert an opposing effect on the maximal force generating capacity in intact myofilaments and thereby obscure an effect of PKCα on maximal force [30].

Phosphorylation of the known PKC sites Ser42/44 and Thr143 is evidently fast as can be judged from the relatively high phosphorylation at these sites in cTn complex incubated with PKCα for less than 1 minute (Figure 5). This implies that rapid alterations in the kinase–phosphatase balance might impact contractile function on a beat–to–beat basis. The MRM assay revealed low endogenous phosphorylation levels of Ser198, which significantly increased after PKCα incubation in both failing tissue and recombinant cTn complex, albeit that the levels reached were low. However, low levels of phosphorylation can exert a significant effect on function [31]. This notion is consistent with the findings of Zhang et al. [28] who observed low levels of Ser198 phosphorylation in healthy control hearts and a significant increased phosphorylation in the failing heart.

In conclusion, the PKCα-induced increase in Ca^2+^-sensitivity of force might be caused by *in vitro* phosphorylation of Thr143 on cTnI or from phosphorylation of our newly identified sites on cTnI and cTnT. Subsequent incubation of the exchanged cardiomyocytes from failing hearts with PKCα decreased the Ca^2+^-sensitivity of force, which is in agreement with our previous study [1] and might either be caused by phosphorylation of Ser42/Ser44 on cTnI, Ser179 on cTnT or by phosphorylation of other myofilamentary target proteins of PKCα.

## Supporting Information

Figure S1 **Cross-phosphorylation of the PKA sites Ser23/Ser24 by PKCα incubation.**
(TIF)

Figure S2 **Exchange of cTn (DD) complex in donor cardiomyocytes.**
(TIF)

Figure S3 **Exchange of endogenous cTn with cTn (DD) complex in non-failing donor tissue.**
(TIF)

Figure S4 **Coomassie stained SDS-PAGE gel of cTn complex.**
(TIF)

Figure S5 **MRM MS traces of PKCα phosphorylated cTnI peptides.**
(TIF)

Figure S6 **Calibration curves of two standard peptides by MRM.**
(TIF)

Table S1 **List of the (synthetic) phosphorylated peptides used for the MRM assay and the corresponding transitions.**
(DOCX)

Table S2 **Data overview of site-specific quantification of PKCα-treated human recombinant cTnI.**
(DOCX)

File S1 **Online Supplement.**
(DOCX)
