# Supplementary figures and images for: PKCα-Specific Phosphorylation of the Troponin Complex in Human Myocardium: A Functional and Proteomics Analysis
Source: PLoS One. 2013 Oct 7;8(10):e74847. doi: 10.1371/journal.pone.0074847 (PMC3792062; doi:10.1371/journal.pone.0074847)

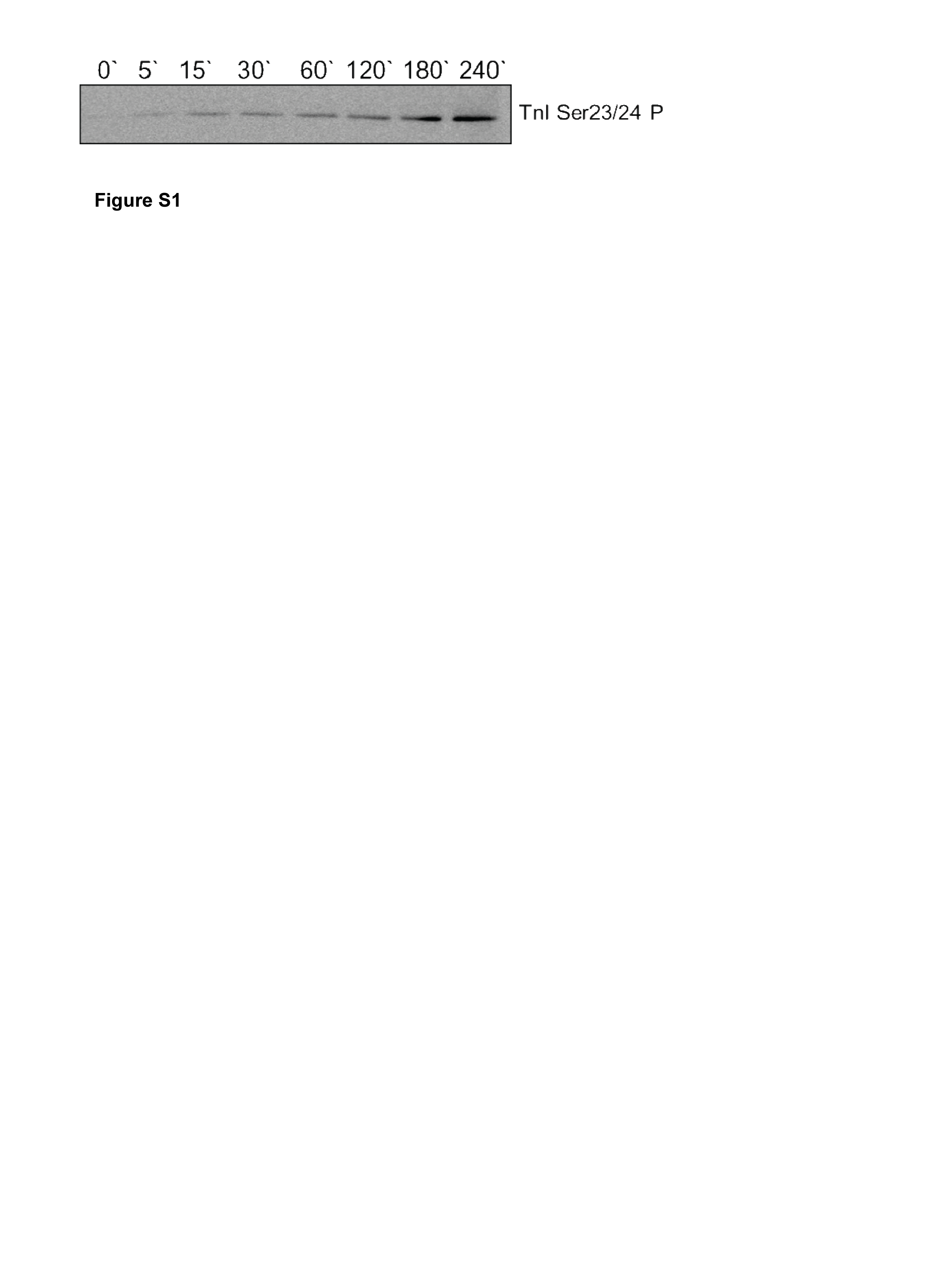

Supplement: Figure S1 — Cross-phosphorylation of the PKA sites Ser23/Ser24 by PKCα incubation. (TIF) [file pone.0074847.s001.tif]

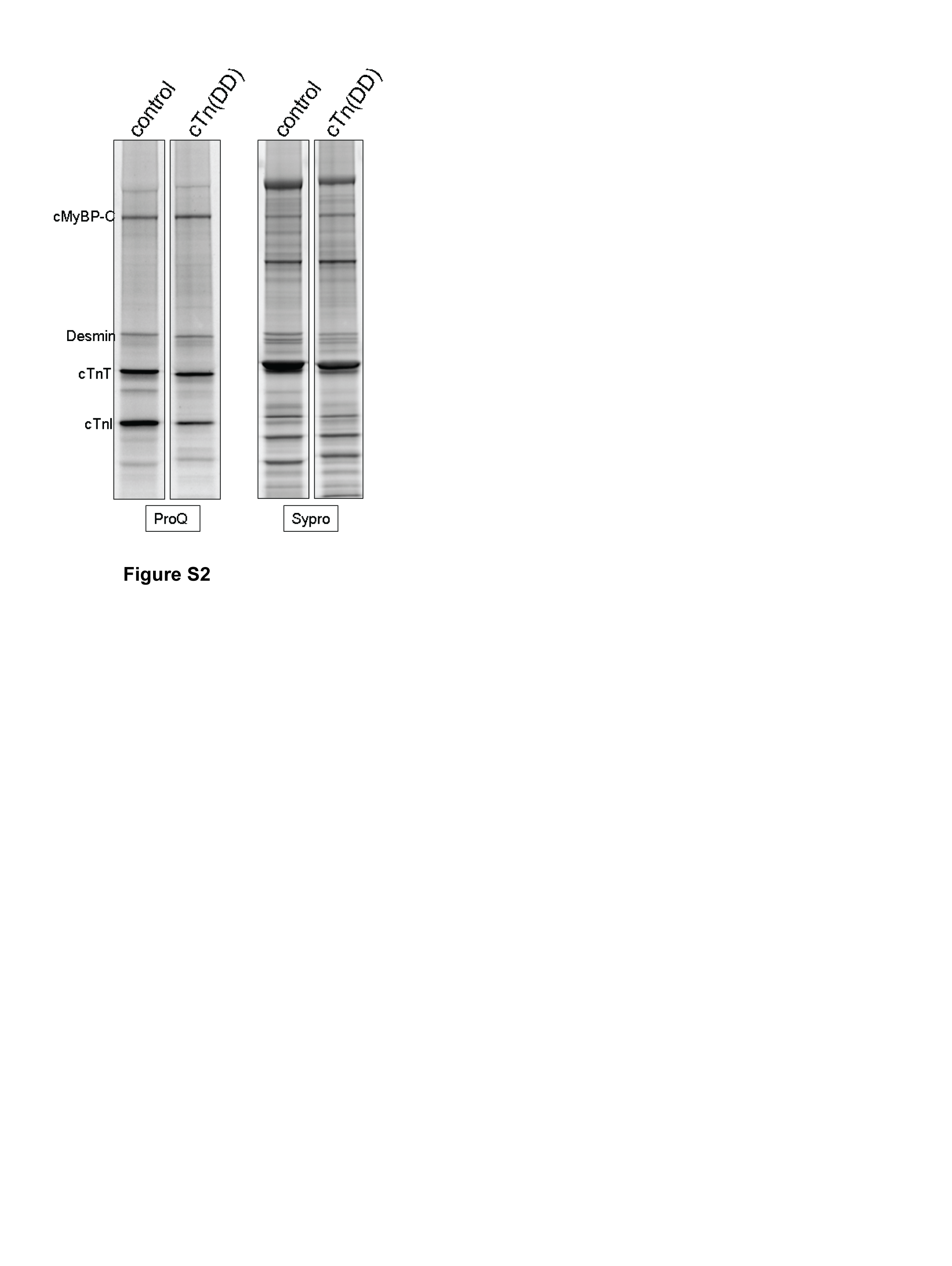

Supplement: Figure S2 — Exchange of cTn (DD) complex in donor cardiomyocytes. (TIF) [file pone.0074847.s002.tif]

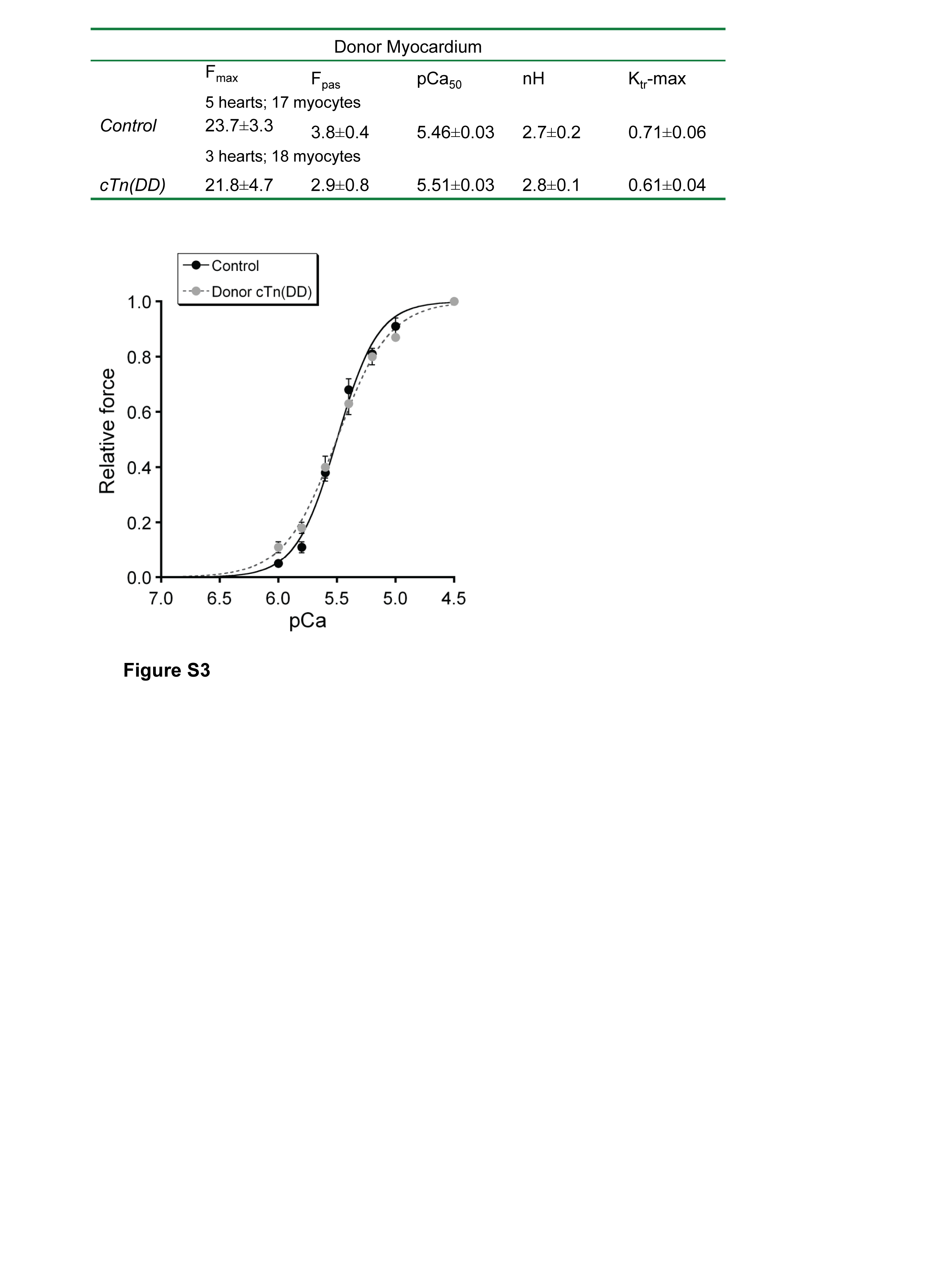

Supplement: Figure S3 — Exchange of endogenous cTn with cTn (DD) complex in non-failing donor tissue. (TIF) [file pone.0074847.s003.tif]

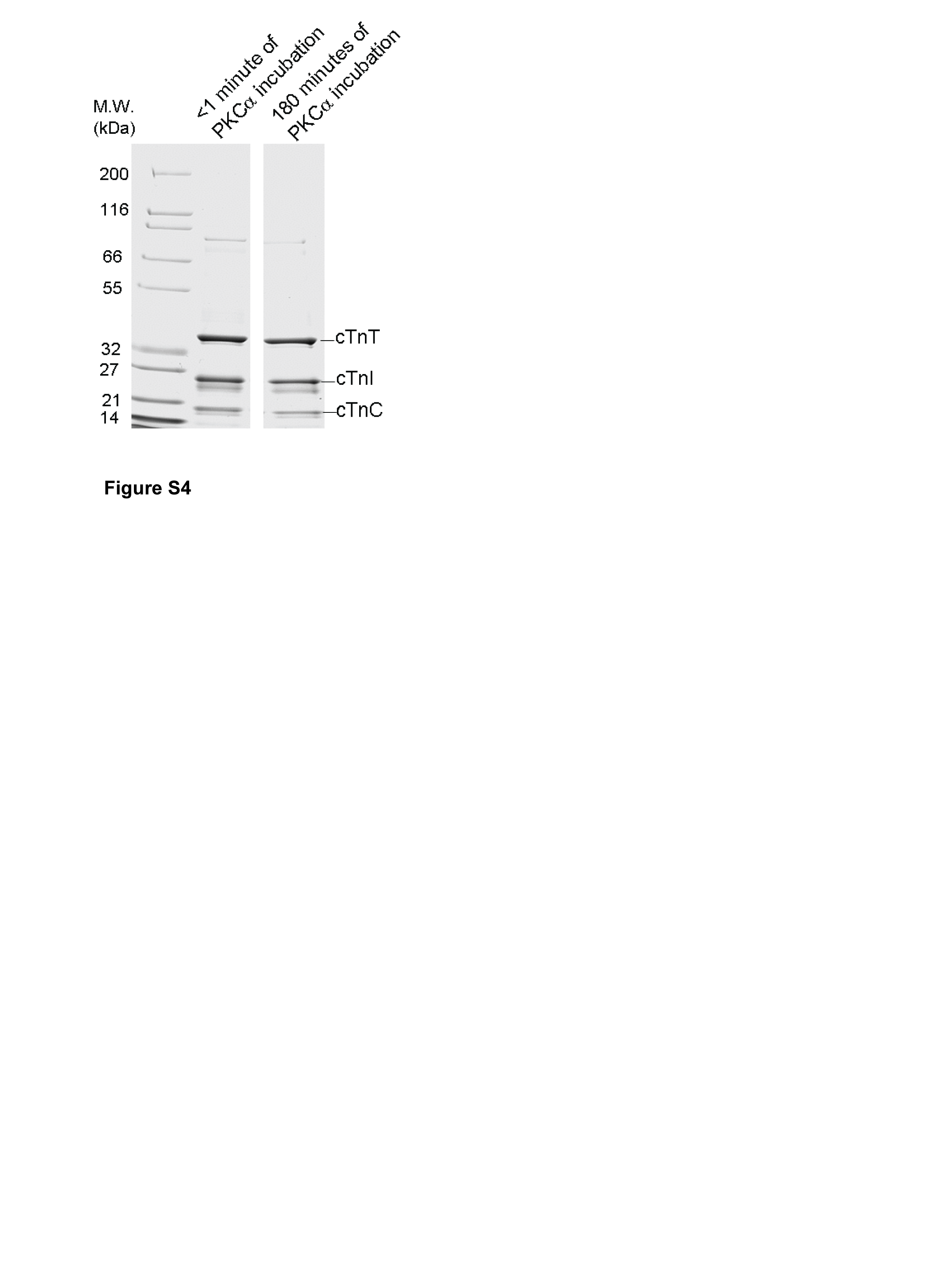

Supplement: Figure S4 — Coomassie stained SDS-PAGE gel of cTn complex. (TIF) [file pone.0074847.s004.tif]

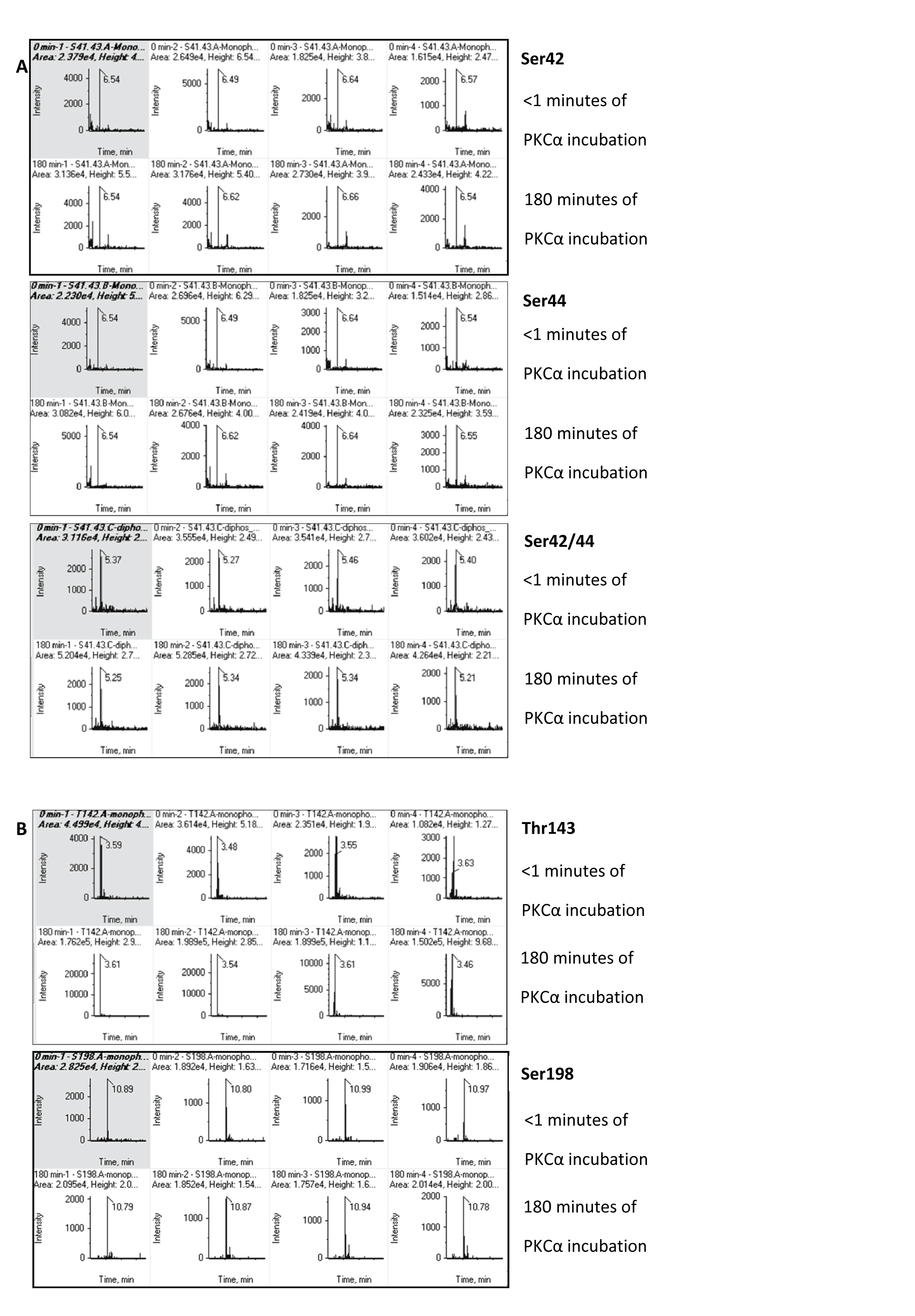

Supplement: Figure S5 — MRM MS traces of PKCα phosphorylated cTnI peptides. (TIF) [file pone.0074847.s005.tif]

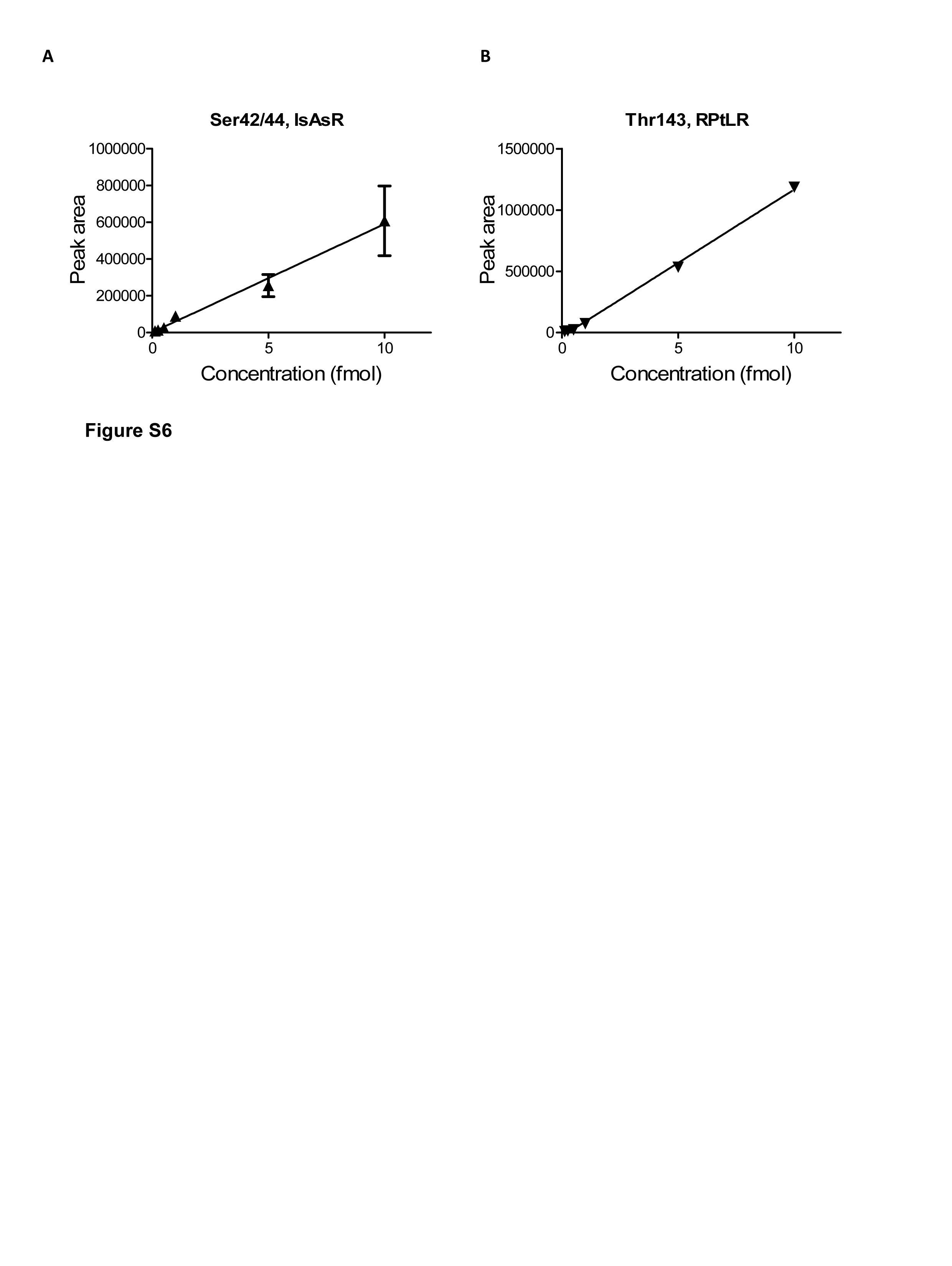

Supplement: Figure S6 — Calibration curves of two standard peptides by MRM. (TIF) [file pone.0074847.s006.tif]
