# Supplementary material for: PKCα-Specific Phosphorylation of the Troponin Complex in Human Myocardium: A Functional and Proteomics Analysis
Source: PLoS One. 2013 Oct 7;8(10):e74847. doi: 10.1371/journal.pone.0074847 (PMC3792062; doi:10.1371/journal.pone.0074847)
Supplement: Table S1 — List of the (synthetic) phosphorylated peptides used for the MRM assay and the corresponding transitions. (DOCX) [file pone.0074847.s007.docx]

| Phospho-site | Q1 | Q3 | Phospho-site | Q1 | Q3 |
| --- | --- | --- | --- | --- | --- |
| Ser42 | 306.9^2+^ | 402.4^1+^ | Ser44 | 307.0^2+^ | 402.3^1+^ |
| IsASR | 306.9^2+^ | 500.5^1+^ | ISAsR | 307.0^2+^ | 258.5^1+^ |
|  | 306.9^2+^ | 333.4^1+^ |  | 307.0^2+^ | 500.4^1+^ |
|  | 306.9^2+^ | 262.3^1+^ |  | 307.0^2+^ | 420.3^1+^ |
|  | 306.9^2+^ | 258.4^1+^ |  | 307.0^2+^ | 333.4^1+^ |
| Ser42/44 | 346.8^2+^ | 412.4^1+^ | Thr143 | 362.1^2+^ | 313.3^1+^ |
| IsAsR | 346.8^2+^ | 298.3^1+^ | RPtLR | 362.1^2+^ | 565.8^1+^ |
|  | 346.8^2+^ | 315.5^1+^ |  | 362.1^2+^ | 468.5^1+^ |
|  | 346.8^2+^ | 482.5^1+^ |  | 362.1^2+^ | 288.4^1+^ |
|  | 346.8^2+^ | 580.2^1+^ |  | 362.1^2+^ | 371.4^1+^ |
| Ser198 | 622.4^2+^ | 829.7^1+^ | Ser198 heavy | 627.4^2+^ | 928.8^1+^ |
| NIDALsGMEGR | 622.4^2+^ | 918.8^1+^ | NIDALsGMEGR* | 627.4^2+^ | 242.4^1+^ |
|  | 622.4^2+^ | 573.7^1+^ |  | 627.4^2+^ | 726.9^1+^ |
|  | 622.4^2+^ | 1016.9^1+^ |  | 627.4^2+^ | 559.6^1+^ |
|  | 622.4^2+^ | 716.8^1+^ |  | 627.4^2+^ | 539.7^1+^ |

**Table S1**
