# Supplementary material for: PKCα-Specific Phosphorylation of the Troponin Complex in Human Myocardium: A Functional and Proteomics Analysis
Source: PLoS One. 2013 Oct 7;8(10):e74847. doi: 10.1371/journal.pone.0074847 (PMC3792062; doi:10.1371/journal.pone.0074847)
Supplement: Table S2 — Data overview of site-specific quantification of PKCα-treated human recombinant cTnI. (DOCX) [file pone.0074847.s008.docx]

**Table S2**

| Phosphosite | Treatment time (minutes) | SDS-PAGE intensity (A.U.) | MRM1 (fmol)/intensity (A.U.) | MRM2  [fmol]/intensity (A.U.) | MRM3  [fmol]/intensity (A.U.) | MRM4 [fmol]/intensity (A.U.) | Average phosphorylation  [fmol]/ intensity  [A.U.] | SEM | P-value |
| --- | --- | --- | --- | --- | --- | --- | --- | --- | --- |
| Ser42 (mono-p) | < 1 | 23.48 | 0.0015 | 0.0016 | 0.0014 | 0.0013 | 0.0015 | 0.00005 |  |
|  | 180 | 21.25 | 0.0018 | 0.0018 | 0.0017 | 0.0017 | 0.0018 | 0.00004* | 0.0002 |
| Ser44 (mono-p) | < 1 | 23.48 | 0.1009 | 0.1062 | 0.0964 | 0.0929 | 0.0991 | 0.0029 |  |
|  | 180 | 21.25 | 0.1221 | 0.1171 | 0.1139 | 0.1127 | 0.1164 | 0.0021* | 0.047 |
| Ser42/44 (di-p) | < 1 | 23.48 | 0.0217 | 0.0249 | 0.0248 | 0.0252 | 0.0241 | 0.0008 |  |
|  | 180 | 21.25 | 0.0406 | 0.0413 | 0.0337 | 0.0331 | 0.0372 | 0.0022* | 0.0172 |
| Ser42/44 (total-p) | < 1 | 23.48 | 0.1241 | 0.1326 | 0.1225 | 0.1194 | 0.1247 | 0.0028 |  |
|  | 180 | 21.25 | 0.1646 | 0.1602 | 0.1493 | 0.1475 | 0.1554 | 0.0042* | 0.0025 |
| Thr143 | < 1 | 23.48 | 0.2679 | 0.2365 | 0.1916 | 0.1465 | 0.2106 | 0.0265 |  |
|  | 180 | 21.25 | 0.8114 | 0.9006 | 0.8652 | 0.7093 | 0.8216 | 0.0416* | 0.0004 |
| Ser198 | < 1 | 23.48 | 0.0031 | 0.0030 | 0.0030 | 0.0030 | 0.0030 | 0.00003 |  |
|  | 180 | 21.25 | 0.0034 | 0.0034 | 0.0033 | 0.0033 | 0.0034 | 0.00001* | 0.0003 |
