# Supplementary material for: PKCα-Specific Phosphorylation of the Troponin Complex in Human Myocardium: A Functional and Proteomics Analysis
Source: PLoS One. 2013 Oct 7;8(10):e74847. doi: 10.1371/journal.pone.0074847 (PMC3792062; doi:10.1371/journal.pone.0074847)
Supplement: File S1 — Online Supplement. (DOCX) [file pone.0074847.s009.docx]

Online Supplement

**Expanded Methods**

Phosphorylation of human recombinant cTn with PKCα and protein analysis

The human cardiac troponin subunits, cTnI, cardiac troponin C (cTnC) and cTnT were expressed and purified as described previously [1]. The troponin subunits were reconstituted into the full troponin complex by mixing the subunits in a 1:1:1 molar ratio. Recombinant cTn complex in which the PKA sites Ser23/24 are mutated into aspartic acid (D) was used to rule out cross-phosphorylation of these sites by PKC, which occurs *in vitro* [2-4]. The pseudo-phosphorylated cTn (cTn(DD)) was expressed and purified using the same protocol as for wild type cTnI, with the minor adjustment that NaCl was omitted from the buffer during binding to the cTnC affinity column. The purified protein was finally dialyzed in 50 mmol/L Tris (pH 7.5), 500 mmol/L NaCl, 1 mmol/L MgCl_2_, 5 mmol/L CaCl_2_ and 2 mmol/L dithiothreitol (DTT).

Cardiac Tn(DD) complex was maximally phosphorylated by human recombinant PKCα isozyme (Sigma, P1782). Thereto, the cTn complex was incubated with PKCα (24 μg/mL PKCα, 1 mmol/L Na_2_ATP, 4 mmol/L MgCl_2_, 6 mmol/L DTT, 10 μmol/L PMA (phorbol 12-myristate 13-acetate; Sigma), 10 μl/ml phosphatase inhibitor cocktail (PhIC, Sigma, P5726), and 5 μl/mL protease inhibitor cocktail (PIC, Sigma, P8340) for 180 minutes at 30°C. Samples were taken at different time–points. The first time-point was taken immediately after all the agents were mixed together. However, during this short time period phosphorylation of cTn could already occur and this time-point was therefore named <1 minute. The phosphorylated cTn(DD) complex (cTn(DD + PKCα) was dialyzed overnight in order to remove ATP.

Analysis of PKCα-mediated phosphorylation of recombinant cTn(DD) complex was assessed on a 4-15% gradient gel (Biorad) stained with ProQ Diamond and Sypro (Molecular Probes). To determine site-specific phosphorylation of cTnI, the gel was blotted (Biorad; semi-dry transfer cell) onto a polyvinylidene difluoride (PVDF) transfer membrane (Hybond) using the protocol supplied by the manufacturer in 1.5 hour at 100mA. Specific monoclonal antibodies against phosphorylated cTnI at sites Ser42 (Abcam; ab59420 dilution 1:2000) and Thr143 (Abcam; ab58546 1:500) in conjunction with a secondary horseradish peroxidase labelled goat-anti-rabbit antibody (DakoCytomation; dilution 1:1000) were used to detect phosphorylated cTnI by chemiluminescence (ECL, Amersham Biosciences).

Exchange of cardiac troponin complex in failing human cardiomyocytes

In the exchange experiments, left ventricular samples (n=6) from end-stage failing idiopathic dilated myocardium (IDCM, NYHA Class IV) were used. In previous studies, we observed that cTnI phosphorylation status in these failing samples is low [5,6].

Single cardiomyocytes were isolated, Triton X-100 permeabilized and exchanged with recombinant cTn complex as described before with a few adjustments [7]. Single cardiomyocytes were mechanically isolated in ice-cold rigor solution (132 mmol/L NaCl, 5 mmol/L KCl, 1 mmol/L MgCl_2_, 10 mmol/L Tris, 5 mmol/L EGTA, 1 mmol/L NaAzide, pH 7.1) and permeabilized by addition of 0.5% Triton X-100 for 5 minutes [8,9]. After permeabilization, cells were washed twice with rigor solution and finally washed in exchange solution (10 mmol/L imidazole, 5 mmol/L MgCl_2_, 3 mmol/L CaCl_2_, 2.5 mmol/L EGTA, pH 6.9). Cardiomyocytes were subsequently incubated overnight at 4°C in exchange solution containing 1.0 mg/mL recombinant human cTn(DD) or cTn(DD + PKCα) complex, 4 mmol/L DTT, 5 μl/mL PIC, 10 μl/mL PhIC, 50 nmol/L CalA (Calyculin A; Sigma) and 100 μmol/L PKC inhibitor (Sigma, P3115). PKC inhibitor was added to block any remaining PKCα activity in order to maintain the endogenous phosphorylation levels. The following day, the cardiomyocytes were washed twice in rigor solution and finally in relaxing solution (5.95 mmol/L Na_2_ATP, 6.04 mmol/L MgCl_2_, 2 mmol/L EGTA, 139.6 mmol/L KCl, 10 mmol/L Imidazole, pH 7.0). Cardiomyocytes kept in exchange solution, without cTn complex added were used to obtain untreated reference values.

Isometric force measurements in single human cardiomyocytes

Force measurements in cardiomyocytes exchanged with cTn complex were performed as described previously [1,9]. Sarcomere length was adjusted to 2.2 μm and force measurements were performed at 15°C. Different pCa (-log_10_[Ca^2+^]) values ranging from 9 (relaxing) to 4.5 (maximally activating) were used to determine the passive force (F_pas_) and maximal force (F_max_), the Ca^2+^-sensitivity of force development (pCa_50_) and the redevelopment of force (K_tr_) after a slack test [10]. To measure the K_tr_, after reaching steady force, the cardiomyocyte was 20% reduced in length within 2 ms and restretched after 30 ms (slack test). During this slack test, force first dropped to zero and after the restretch quickly redeveloped to the original steady state level. A single exponential was fitted to estimate the rate constant of force redevelopment at maximal activation (Ktr-max).

Active force was obtained by subtracting passive force from the total force, i.e., F_active_ = F_total_ – F_passive_. After maximal activation, force measurements were performed at submaximal [Ca^2+^] to determine myofilament Ca^2+^-sensitivity. At the end of these measurements the cell was again maximally activated to assess the decline in force with time. When the control force at the end of the experiment was less than 80% of the previous measurement the myocyte was excluded from the study. After these initial force measurements, myocytes were incubated for 60 minutes at 20°C in pCa 6.0 solution with 10 μg/mL PKCα, 10 μmol/L PMA, 6 mmol/L DTT and 50 nmol/L CalA added [6]. After these incubations, force measurements were repeated. Control measurements in which the force/pCa relationship was determined twice in each myocyte indicated that ‘run-down’ of the cardiomyocytes did not affect the force/pCa relationship [4,11].

LC MS/MS analysis of human recombinant cTn complex incubated with PKCα

In-gel digestion

Coomassie-stained protein bands were excised and processed for in-gel digestion [12]. Briefly, protein bands were washed in 50 mmol/L ammonium bicarbonate (ABC, pH 7.9) and dehydrated three times in 50 mmol/L ABC and 50% acetonitrile (ACN). Subsequently, cysteine bonds were reduced with 10 mmol/L DTT for one hour at 56°C and alkylated with 50 mmol/L iodoacetamide for 45 minutes at room temperature in the dark. After two subsequent wash/dehydration cycles the protein bands were dried 10 min in a vacuum centrifuge (ThermoFisher, Breda, the Netherlands) and incubated overnight with 6.25 ng/µL trypsin in 50 mmol/L ABC at 25°C. Peptides were extracted once in 100 µL 1% formic acid and subsequently two times in 100 µL 50% ACN in 5% formic acid. The volume was reduced to 50 µl in a vacuum centrifuge prior to LC-MS/MS analysis.

*LC-MS/MS analysis*

Peptides were separated using an Ultimate 3000 nanoLC system (Dionex LC-Packings, Amsterdam, The Netherlands) equipped with a 20 cm x 75 µm ID fused silica column custom packed with 3 µm 120 Å ReproSil Pur C18 aqua (Dr Maisch GMBH, Ammerbuch-Entringen, Germany). After injection, peptides were trapped at 6 µL/min on a 20 mm x 100 µm ID precolumn packed with 5 µm 120 Å ReproSil Pur C18 aqua at 2% buffer B (buffer A: 0.05% formic acid in water buffer B: 80 % ACN + 0.05% formic acid in water) and separated at 300 nL/min in a 10-40% buffer B gradient in 60 min. Eluting peptides were ionized on-line at 1.7 kV in a Nanomate Triversa Chip-based nanospray source using a Triversa LC coupler (Advion, Ithaca, NJ). Intact peptide mass spectra and fragmentation spectra were acquired on a LTQ-FT hybrid mass spectrometer (Thermo Fisher, Bremen, Germany). Intact masses were measured at a resolution of 50,000 in the ICR cell using a target value of 1 x 10^6^ charges. In parallel, following an FT pre-scan, five highest-intensity precursor-ion signals (charge-states 2^+^ and higher) were submitted to MS/MS in the linear ion trap (3 amu isolation width, multistage activation, 30 ms activation, 35% normalized activation energy, Q value of 0.25 and a threshold of 5,000 counts). Dynamic exclusion was applied with a repeat count of 1 and an exclusion time of 30 sec.

Database searching

MS/MS spectra were searched against the human IPI database 3.48 (71401 entries) using Sequest (version 27, rev 12), which is part of the BioWorks 3.3 data analysis package (Thermo Fisher, San Jose, CA). MS/MS spectra were searched with a maximum allowed deviation of 10 ppm for the precursor mass and 1 amu for fragment masses. Methionine oxidation, serine, threonine and tyrosine phosphorylation and cysteine carboxamido­methylation were allowed as variable modifications, two missed cleavages were allowed and the minimum number of tryptic termini was 1. After database searching the data files were imported into Scaffold 2.01 (Proteomesoftware, Portland, OR). Scaffold was used to organize the data and to validate peptide identifications using the PeptideProphet [13] algorithm. Only identifications with a probability >95% were retained. Subsequently, the ProteinProphet [14] algorithm was applied and protein identifications with a probability of >99% with 2 peptides or more in at least one of the samples were retained [13,15]. Proteins that contained similar peptides and could not be differentiated based on MS/MS analysis alone were grouped.

**MRM MS assay of PKCα-treated donor and failing tissue and human recombinant cTn**

*Assay development*

The incubation of donor and failing tissue with PKCα was performed as described before [6]. 50 *µ*g of myofilament protein (with and without PKCα treatment) mixture was separated by NuPAGE gel electrophoresis and gel band bands were excised and processed for in-gel digestion as described above. Digested protein samples were analyzed using a 4000 QTRAP hybrid triple quadrupole/linear IT mass spectrometer (Applied Biosystems, Foster City, CA, USA) operating in positive ion mode. All raw data was calibrated by synthesized internal standard peptides (total cTnI: NITEIADLTQK, Ser198 phosphorylated peptide: NIDALsGMEGR) as described below, then normalized to total troponin I. This provided the the stoichiometric quantity (fmol phosphorylation/ fmol protein) of phosphorylation of Ser198 on cTnI Control (untreated failing tissue) and PKCα-treated tissue from heart failure patients (*n* = 4 per group; technical replicates = 3). Data is shown as fold increase.

To quantify the phosphorylation status of each site in PKCα-treated human recombinant cTn complex, a total of 40 MRM assays (with 5 transitions per peptide) were developed for the phosphorylated versions of the tryptic peptides containing the various potential modified residues. Online Table I lists the peptides and their corresponding transitions composed of specific pairs of Q1/Q3 ions. The peptides were synthesized by New England Peptide. Peptide NIDALsGMEGR* has a N15 incorporated at the C-terminus (Online Table I). Amino acid analysis was carried out to determine the concentration of each synthetic peptide. Each MRM was optimized for maximum sensitivity which is dependent on the setting of the instruments parameters including declustering potential (DP), collision energy (CE), and collision cell exit potential (CXP). Each sample was run in quadruplicate.

*Standard curves*

The standard curves were obtained by measuring the synthesized internal standard phosphorylated peptides IsASR, ISAsR, IsAsR, RPtLR, and NIDALsGMEGR in a six-point dilution. These peptides were spiked into a mixture containing 10 heavy labeled peptides of cTnI at known concentrations (fixed at 1 fmol/μL) to mimic a digest matrix of human cTnI. LC-MRM analysis was performed on the triple quadrupole mass spectrometer Qtrap4000 using the optimized parameters described above. Each standard curve for the above phosphorylated peptides was then generated from precise quantities of each of these internal standard peptides at 0.125, 0.25, 0.5, 1, 5, and 10 fmol/μL in triplicates.

*Sample quantification*

Digested protein samples were analyzed using a 4000 QTRAP hybrid triple quadrupole/linear IT mass spectrometer (Applied Biosystems, Foster City, CA, USA) operating in positive ion mode. Peptides were separated by nanoflow LC using an Eksigent nano- LC system (Eksigent Technologies, Dublin, CA, USA). A PicoFrit C18 column, 75μm x 100mm) using a linear AB gradient (Buffer A: 0.1%FA/2%ACN/98%H_2_O, Buffer B: 0.1%FA/98%ACN/2%H_2_O) at a flow rate of 500nL/min. The gradient consisted of 5% Buffer B increasing to 40% Buffer B over 25 min. Each sample was run in quadruplicate.

Data analysis

*Myofilament data analysis*:

Myofilament data analysis was performed using the modified Hill equation to fit force-pCa relations: F(Ca^2+^)/F_0_= [Ca^2+^]^nH^/(Ca_50_^nH^+ [Ca^2+^]^nH^), where F is steady-state force, F_0_ the steady-state force at saturating [Ca^2+^], nH a measure of the steepness of the relationship and pCa_50_ represent the midpoint of the relation. K_tr_ was determined from an exponential curve fit of force redevelopment after a slack test, consisting of a rapid (<1 ms) shortening of the myocyte by 20% of its initial length, followed after 30 ms by a rapid restretch to the original length. Comparisons between the cTn(DD)and cTn(DD + PKCα) groups were analyzed using an unpaired Student *t*-test. Values are given as means ± S.E.M. of *n* myocytes.

*MRM Data analysis:*

MRM data was analyzed using the Applied Biosystems/MDS Sciex Analyst software 1.5 for data acquisition and processing. Multiquant 1.0 was used for data analysis. Each transition was manually inspected to ensure the correct peak was quantified and baseline was appropriate.

Total amount of cTnI in each cTn sample was independently determined based on data of N15 labeled peptides and unlabeled peptides. The acquired average peak areas were normalized to a relative phosphosite-specific level (fmol), based on the standard curves (Online Figure V). The obtained values were corrected by the intensity of the SDS-PAGE gel bands for protein loading (Online Table II, Online Figure 1). There was 30 µg of recombinant cTn complex loaded on the SDS-PAGE gel for each time-point.

Legends

Table S1: List of the (synthetic) phosphorylated peptides used for the MRM assay and the corresponding transitions. Each transition was optimized, as described in the expanded methods. Peptide NIDALsGMEGR* has a N15 incorporated at the C-terminus. Abbreviations: Ser, serine; Thr, threonine; Q1, parental ions; Q3, fragment ions*.* 1+ and 2+ means the charge state of the peptide.

Table S2: Data overview of site-specific quantification of PKCα-treated human recombinant cTnI.

Technical replicates were 4: MRM1 to MRM4. Abbreviations: p, phosphorylation; SDS-PAGE, sodium dodecyl sulphate-polyacrylamide gel electrophoresis; MRM, multiple reaction monitoring; A.U., arbitrary units; SEM, standard error of the mean; Ser, serine; Thr, threonine. * P<0.05 in a paired two-tailed student *t*-test (< 1 vs. 180 minutes of PKCα incubation).

**Figure S1: Cross-phosphorylation of the PKA sites Ser23/Ser24 by PKCα incubation.** Recombinant cTn complex was incubated with PKCα. Samples were taken at different time points. Western blot analysis showed cTnI phosphorylation at PKA sites Ser23 and Ser24 (Antibody 8i-7 Spectral Diagnostics).

Figure S2: Exchange of cTn(DD) complex in donor cardiomyocytes. ProQ Diamond analysis of cTn(DD) exchange in donor control cardiomyocytes showed a level of 69.4±13.3% (n=3) exchange. The percentage of cTn complex exchange was based on the reduction in cTnI phosphorylation after cTn(DD) exchange as cTn(DD) is not stained by the phosphor-specific ProQ Diamond stain.

Figure S3: Exchange of endogenous cTn with cTn(DD) complex in non-failing donor tissue. Cardiomyocytes exchanged with cTn(DD) complex showed no functional differences compared to control cardiomyocytes in which no cTn complex was present during the overnight exchange protocol. It can be seen that exchange of cTn(DD) in donor cells has hardly any effect on force development and its Ca^2+^-sensitivity. This lack of effect of pseudophosphorylated cTn(DD) is as expected since non-failing donor samples show high cTnI phosphorylation at the PKA sites (Ser23/24) [7].

## Figure S4: Coomassie stained SDS-PAGE gel of cTn complex. Cardiac Tn complex was incubated with PKCα for <1 min or 180 min and separated on a coomassie stained SDS-PAGE gel. Samples were run on the same gel. Cardiac TnI bands were excised from the gel for the MRM analysis. The densitometry of the cTnI bands were used to calculate the final concentration of phosphorylation (Figure 3B, Table S2).

## Figure S5: MRM MS traces of PKCα phosphorylated cTnI peptides. LC elution profiles for the tryptic peptides of PKCα treated (<1 and 180 min) cTnI. The peaks for cTnI phosphorylation on site Ser42, Ser44, the diphosphorylated sites Ser42/44 (A), Thr143 and the novel site Ser198 (B) are shown. Ser42 and Ser44 have retention times of approximately 6.54 min, Ser42/44 of approximately 5.37 min, Thr143 of approximately 3.59 min and Ser198 of approximately 10.89 min. Each peptide is analyzed based on their Q1/Q3 transitions. The different boxes represent the LC elution profiles in quadruplicate for the peptides containing the phosphorylation sites Ser42, Ser44, Ser42/44, Thr143 and Ser198. Ser (S), serine; Thr (T), threonine; monophos, monophosphorylation; diphos, diphosphorylation.

## Figure S6: Calibration curves of two standard peptides by MRM. Two representative calibration curves of the standard peptides for residues Ser42/44 (A) and Thr143 (B). Amino acid analysis was performed on the peptides to obtain the correct concentration. Peak areas were measured in triplicate at different concentrations of the standard peptides. The calibration curves were used to calculate the concentration of phosphorylation peptides in the samples.

Reference List

1. Narolska NA, Piroddi N, Belus A, Boontje NM, Scellini B et al. (2006) Impaired diastolic function after exchange of endogenous troponin I with C-terminal truncated troponin I in human cardiac muscle. Circ Res. 99:1012-1020.

2. Kobayashi T, Yang X, Walker LA, Van Breemen RB, Solaro RJ (2005) A non-equilibrium isoelectric focusing method to determine states of phosphorylation of cardiac troponin I: identification of Ser-23 and Ser-24 as significant sites of phosphorylation by protein kinase C. J Mol Cell Cardiol. 38:213-218.

3. Swiderek K, Jaquet K, Meyer HE, Schachtele C, Hofmann F et al. (1990) Sites phosphorylated in bovine cardiac troponin T and I. Characterization by 31P-NMR spectroscopy and phosphorylation by protein kinases. Eur J Biochem. 190:575-582.

4. van der Velden J, Narolska NA, Lamberts RR, Boontje NM, Borbely A et al. (2006) Functional effects of protein kinase C-mediated myofilament phosphorylation in human myocardium. Cardiovasc Res. 69:876-887.

5. Hamdani N, Kooij V, van Dijk S, Merkus D, Paulus WJ et al. (2008) Sarcomeric dysfunction in heart failure. Cardiovasc Res. 77:649-658.

6. Kooij V, Boontje N, Zaremba R, Jaquet K, dos Remedios CG et al. (2010) Protein kinase C alpha and epsilon phosphorylation of troponin and myosin binding protein C reduce Ca2+ sensitivity in human myocardium. Basic Res Cardiol. 105:289-300.

7. Kooij V, Saes M, Jaquet K, Zaremba R, Foster DB et al. (2010) Effect of troponin I Ser23/24 phosphorylation on Ca(2+)-sensitivity in human myocardium depends on the phosphorylation background. J Mol Cell Cardiol. 48:954-963.

8. Hofmann PA, Miller WP, Moss RL (1993) Altered calcium sensitivity of isometric tension in myocyte-sized preparations of porcine postischemic stunned myocardium. Circ Res. 72:50-56.

9. van der Velden J, Klein LJ, van der BM, Huybregts MA, Stooker W et al. (1998) Force production in mechanically isolated cardiac myocytes from human ventricular muscle tissue. Cardiovasc Res. 38:414-423.

10. van der Velden J, Boontje NM, Papp Z, Klein LJ, Visser FC et al. (2002) Calcium sensitivity of force in human ventricular cardiomyocytes from donor and failing hearts. Basic Res Cardiol. 97 Suppl 1:I118-I126.

11. van der Velden J, Papp Z, Boontje NM, Zaremba R, de Jong JW et al. (2003) The effect of myosin light chain 2 dephosphorylation on Ca2+ -sensitivity of force is enhanced in failing human hearts. Cardiovasc Res. 57:505-514.

12. Gundry RL, White MY, Murray CI, Kane LA, Fu Q et al. (2009) Preparation of proteins and peptides for mass spectrometry analysis in a bottom-up proteomics workflow. Curr Protoc Mol Biol. Chapter 10:Unit10.

13. Keller A, Nesvizhskii AI, Kolker E, Aebersold R (2002) Empirical statistical model to estimate the accuracy of peptide identifications made by MS/MS and database search. Anal Chem. 74:5383-5392.

14. Nesvizhskii AI, Keller A, Kolker E, Aebersold R (2003) A statistical model for identifying proteins by tandem mass spectrometry. Anal Chem. 75:4646-4658.

15. Shevchenko A, Wilm M, Vorm O, Mann M (1996) Mass spectrometric sequencing of proteins silver-stained polyacrylamide gels. Anal Chem. 68:850-858.
